# Bacteriophages of wastewater foaming-associated filamentous *Gordonia* reduce host levels in raw activated sludge

**DOI:** 10.1038/srep13754

**Published:** 2015-09-09

**Authors:** Mei Liu, Jason J. Gill, Ry Young, Elizabeth J. Summer

**Affiliations:** 1 Ecolyse Inc., 11142 Hopes Creek Rd., College Station, Texas 77845, USA; 2 Center for Phage Technology, 2128 TAMU, Texas A&M University, College Station, TX 77843, USA; 3 Department of Animal Science, 2471 TAMU, Texas A&M University, College Station, TX 77843, USA; 4 Department of Biochemistry and Biophysics, 2128 TAMU, Texas A&M University, College Station, TX 77843, USA

## Abstract

Filamentous bacteria are a normal and necessary component of the activated sludge wastewater treatment process, but the overgrowth of filamentous bacteria results in foaming and bulking associated disruptions. Bacteriophages, or phages, were investigated for their potential to reduce the titer of foaming bacteria in a mixed-microbial activated sludge matrix. Foaming-associated filamentous bacteria were isolated from activated sludge of a commercial wastewater treatment plan and identified as *Gordonia* species by 16S rDNA sequencing. Four representative phages were isolated that target *G. malaquae* and two un-named *Gordonia* species isolates. Electron microscopy revealed the phages to be siphophages with long tails. Three of the phages - GordTnk2, Gmala1, and GordDuk1 - had very similar ~76 kb genomes, with >93% DNA identity. These genomes shared limited synteny with *Rhodococcus equi* phage ReqiDocB7 and *Gordonia* phage GTE7. In contrast, the genome of phage Gsput1 was smaller (43 kb) and was not similar enough to any known phage to be placed within an established phage type. Application of these four phages at MOIs of 5–15 significantly reduced *Gordonia* host levels in a wastewater sludge model by approximately 10-fold as compared to non-phage treated reactors. Phage control was observed for nine days after treatment.

The activated sludge process involves the biological conversion of solids and pollutants and is an established method for sewage and industrial wastewater treatment worldwide^1^,^2^. Activated sludge, the essential component of this process, includes a mixed and variable consortium of micro- and macro-organisms including viruses, bacteria, protozoa and metazoa who function in the transformation of organic materials into a liquid mixture that is relatively low in suspended solids and organic compounds^1^,^3^. Among the key organic degraders, filamentous bacteria bond to other floc-forming organisms with biopolymers and provide important floc “backbones” to support the structure and shape of compact flocs for efficient sludge settling^2^. The excessive growth of filamentous bacteria, however, trap and stabilize air bubbles when combined with biosurfactants, resulting in stable sludge foaming and biomass bulking on the surface of aerated reactors. Sludge foaming is a frequent operational problem and prevents adequate flocculation and impedes proper sludge mass settling^1^,^4^. Filamentous bacteria associated with activated sludge and foaming have been extensively characterized based on filament morphotypes^5^,^6^,^7^, fluorescence *in situ* hybridization using rRNA-targeted probes^8^,^9^,^10^,^11^, and high-throughput deep-sequencing-based population analysis^12^,^13^,^14^,^15^. Accumulated studies revealed the diverse compositions of filamentous bacteria involved in foaming, and suggested the common roles of long unbranched filaments of *Candidatus* ‘*Microthrix parvicella*’ or short branched filaments of mycolic acid-containing actinomycetes, or Mycolata^3^,^16^. Mycolata associated with sludge foaming include the genera *Corynebacterium*, *Dietzia*, *Gordonia*, *Mycobacterium*, *Nocardia*, *Rhodococcus*, *Skermania*, *Tsukamurella*, and *Williamsia*^17^. *Gordonia* is frequently reported to be the predominant genus within the foaming-associated mycolata^18^,^19^, although the high abundance of *Gordonia* is not the sole determinant of a foaming event^15^.

Current control methods for sludge foaming reply on physio-chemical methods, including return sludge flow rate and aeration manipulation, surfactants and chlorine addition. These efforts are not always effective and run the risk of secondary perturbations to the sustainable sludge process^20^. An alternative treatment approach would be the use of bacteriophage, is capable of specifically targeting the foam causing organisms without causing collateral damage to other microorganisms^21^,^22^. Phages are a normal component of the wastewater environment^23^,^24^. The idea of using phages as antimicrobial agents, dating back to the earliest days of phage research, is an area of recently renewed interest^25^,^26^. Most of the recent research in phage-based antimicrobials has focused on pathogens of medical or agricultural importance^27^,^28^. The use of phages for the control of problem bacteria in environmental and/or industrial settings also has great potential in replacing inefficient and often environmentally harmful biocides.

Phages against filamentous bacteria, especially different species of mycolata, have been isolated and their therapeutic applications documented in several studies. Mycobacteria species in particular have been the subject of focused efforts^29^,^30^. Multiple phages against *Rhodococcus equi* were characterized and demonstrated to be capable of reducing *R. equi* load in a soil matrix^31^. Phages isolated from a wastewater treatment plant were shown to have promising potential in controlling biomass bulking caused by the filamentous bacteria *Haliscomenobacter hydrossis*^32^. Sludge – associated Mycolata hosts for which phages have been identified include *Tsukamurella* spp., *Gordonia*, *Rhodococcus*, *Nocardia*^22^,^33^,^34^,^35^. Two of these phages - GTE2 and GTE7 - were demonstrated to reduce their host level and prevent stable foam production by their host bacteria in pure laboratory cultures tested in a laboratory foaming apparatus^33^,^36^. Beyond these promising results, the next step in the development of phage-based foam control method is to determine if phage can control foaming associated bacteria directly in the complex chemical and microbial environment of the activated sludge matrix. Additionally, the value of a phage for bacterial control needs to be determined in reference to how broad each phage host range is against wastewater treatment plant-associated hosts. Here, filamentous bacteria were cultured from several wastewater treatment plant foaming outbreaks, and used to isolate cognate phages capable of controlling these bacteria. The isolated phages were evaluated for use in reducing *Gordonia* levels in raw activated sludge.

## Results

### Isolation of *Gordonia* from foaming activated sludge

**Figure 1:**
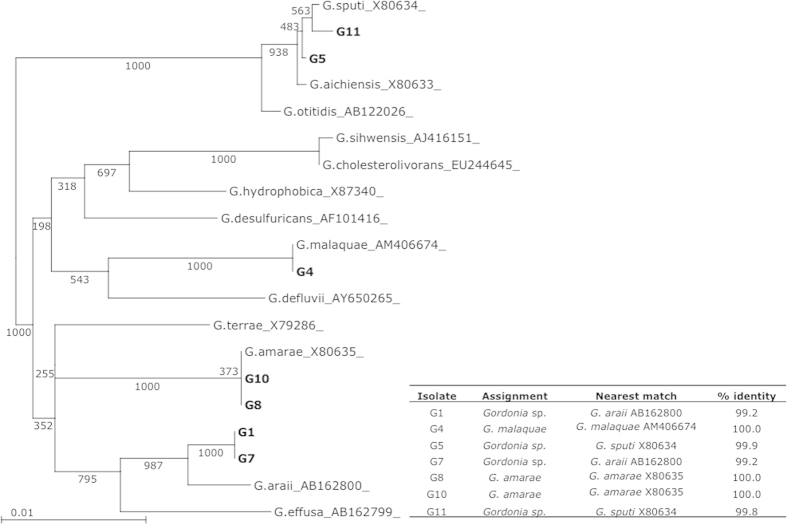
Phylogenetic tree derived from 16S rDNA gene sequences, created using the neighbour-joining method with gap positions excluded. Numbers on the tree indicate bootstrap percentages (from 1000 replicates) for branch points. Assignments of identities of the *Gordonia* isolates are indicated. Strains G5 and G11 were highly similar to *G. sputi*, *G. aichiensis* and *G. otitidis* and thus were identified to the genus level only.

Activated sludge foam samples were collected from aeration basins from a local wastewater treatment plant. Phase contrast microscopic examination revealed that the samples contained compact flocs of intermeshing, true branching filamentous organisms (Supplementary Fig. S1), a typical morphological feature associated with nocardioform bacteria^1^. The foam samples were plated onto both semi-selective and non-selective agar plates for filamentous bacteria isolation and purification. Seven isolates exhibiting filamentous cell growth morphologies (formed short hyphae) during colony development were collected (Supplementary Fig. S1). All isolates developed colonies with irregular margins and appeared white to beige at the beginning of incubation. Strain G1 produced a pink pigment, and G5 and G11 produced yellow and orange/red pigment, respectively, over prolonged incubation. Analysis of the 16s rDNA sequences (~1200 bp) of these isolates revealed four clusters, suggesting four taxonomical groups. By comparing to bacterial type strains in the database of the Ribosomal Database Project (rdp.cme.msu.edu), a phylogenetic tree was generated (Fig. 1). All seven isolates were identified at the genus level as *Gordonia*. Identification to the species level was suggested in the case of isolates G4 (*G. malaquae*), G8 and G10 (*G. amarae*). The 16S sequences of G5 and G11 were highly similar (>99.5%) to *G. sputi, G. aichiensis* and *G. otitidis,* and so could only be confidently identified to the genus level. The remaining two isolates, G1 and G7, were also identified only to the genus level due to their closest match (*G. araii*) being below the desired 99.5% cutoff. The sequenced regions of isolates G1 and G7 were identical to each other and most similar to *G. araii* (Fig. 1). Species designation solely based on 16sDNA sequences is limited and its accuracy needs to be confirmed by further phenotypical characterization.

### Isolation and characterization of *Gordonia* phages

**Figure 2:**
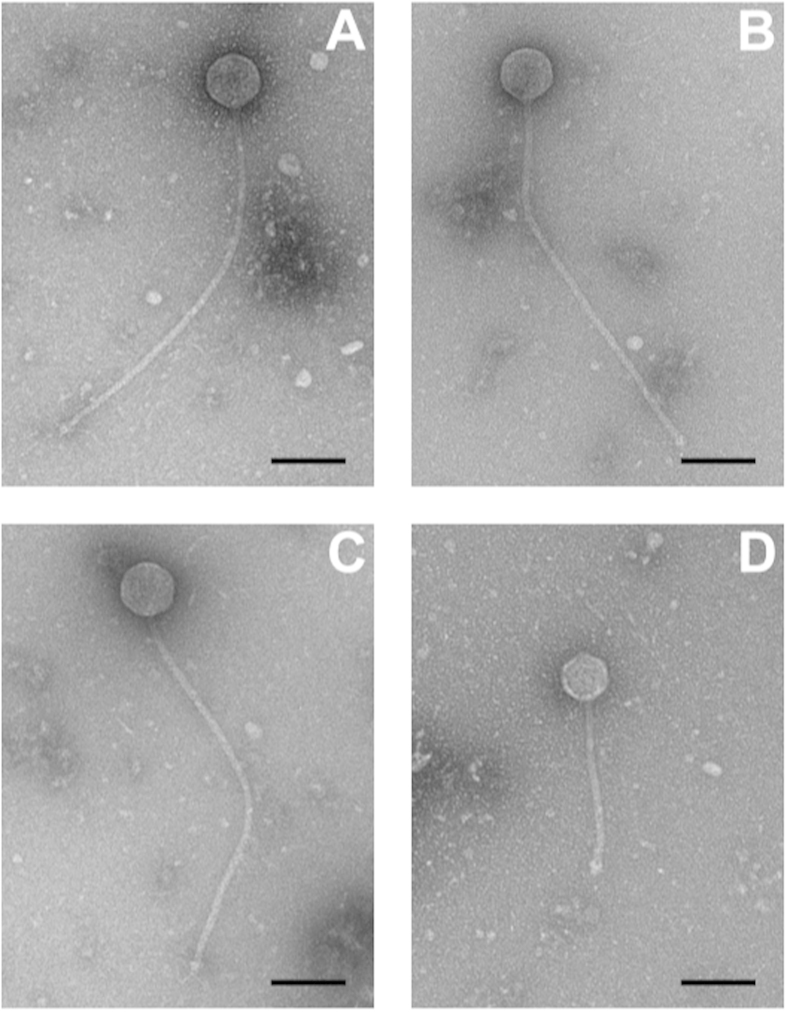
Transmission electron micrographs of phages (A) GordTnk2, (B) Gmala1, (C) GordDuk1, and (D) Gsput1. Samples were stained with 2% (w/v) uranyl acetate and observed at 100 kV. Scale bar is 100 nm.

Fourteen phages were isolated from wastewater using a panel of the seven *Gordonia* strains described above for phage enrichments. The host range of each phage was tested at its routine test dilution, and phages were found to form plaques on 4 of the 7 strains (Table 1). Based on host range, the seven phages could be grouped into four groups, of which only groups 1 and 2 were capable of attacking more than one of the strains (Table 1). Of the strains in the panel, *Gordonia* sp. strain G7 was the most susceptible, being sensitive to phages of groups 1–3. The phages within each host range group generated identical patterns based on restriction fragment length polymorphism (RFLP) using the enzymes EcoRI, BamHI, and HindIII (data not shown). GordTnk2, Gmala1, GordDuk1, and Gsput1, were picked to represent the four phage groups for further testing. The morphologies of the four phages were determined by TEM (Fig. 2). All four phages possessed icosahedral capsids and long, non-contractile tails of siphophages. Three phages, GordTnk2, GMala1, GordDuk1 shared similar average head diameters (72–75 nm) and extremely long tail lengths (~520 nm). Phage Gsput1 was significantly smaller, with head diameter and tail length of 62 ± 1 nm and 229 ± 5 nm, respectively (Fig. 2 and Table 2).

### Genomes of GordTnk2, Gmala1, and GordDuk1

**Figure 3:**
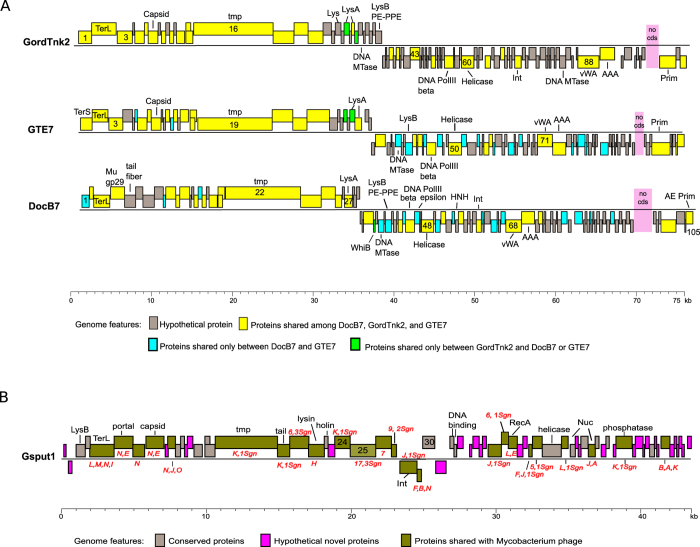
Genome map of Gordonia phages GordTnk2 and Gsput1. GordTnk2 is presented to represent Gmala1 and GordDuk1, as these three phages share great similarity in genome arrangement. Predicted genes are represented by boxes above and below the heavy black line; boxes above the lines are genes encoded on the forward strand, and those below the lines are on the reverse strand. The ruler below the genomes indicates the scale (in kb). Genome features are color coded according to the legend. (**A**) Comparison map of GordTnk2 to *Gordonia* phage GTE7 (accession No. NC_016166), and *Rhodococcus equi* phage ReqiDocB7 (accession No. NC_023706). Proteins sharing identities (e value < 10^−5^) among three phages were identified and color coded according to the legend. (**B**) Genome map of Gsput1. Proteins sharing identities (e value < 10^−5^) with Mycobacterium phage were identified and the clusters or singletons (Sgn) of the Mycobacterium phage are indicated in red letters. Abbreviations: TerS, terminase small subunit; TerL, terminase large subunit; tmp, tape measure protein; Prim, primase; Pol, DNA polymerase; DNA MTase, DNA methylase; HNH, homing endonuclease; Int, integrase; vWA, Von Willebrand factor type A domain protein; AAA, ATP-hydrolyzing domain protein; RecA, recombinase; Nuc, nuclease.

**Figure 4:**
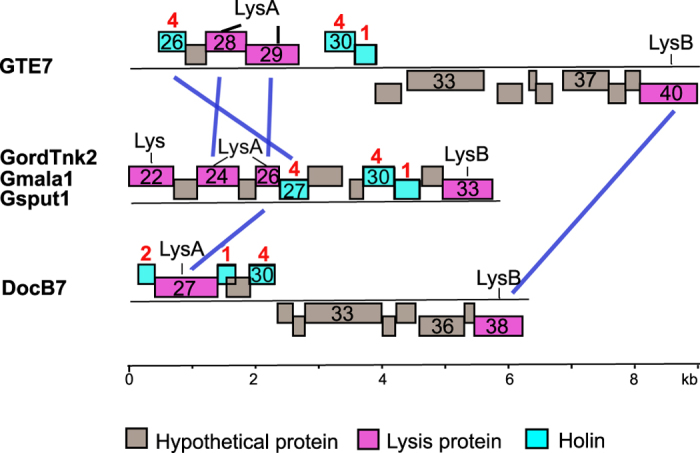
Lysis genes in *Gordonia* phages GordTnk2, Gmala1, and GordDuk1, and their comparisons to *Gordonia* phage GTE7 and *Rhodococcus equi* phage ReqiDocB7. Predicted genes are represented by boxes above and below the black line (encoded on the forward and reverse strand, respectively). The ruler below the genes indicates the scale (in kb). Genome features are color coded according to the legend. Lysis and holin proteins sharing identities (e value < 10^−5^) among presented phages were linked with blue lines. The number of predicted transmembrane domains in holin is indicated above the protein.

The genomic DNA of the four *Gordonia* phages were sequenced (Supplementary Tables S1, S2, S3, and S4). Consistent with their similar morphologies, phages GordTnk2, Gmala1 and GordDuk1 were quite similar in genome size (75 ~ 76 kb, encoding 90–98 proteins), with high overall DNA identity (93%–95%) (Table 2). All three phage genomic termini were determined to be pac-type with limited circular permutation, based on a combination of restriction mapping and analysis of the shotgun assembly, which resolved into a single circularly permuted contig (data not shown). The core genome of phages GordTnk2, Gmala1, and GordDuk1 consisted of 90 protein-coding genes present in all three genomes. Gene-content differences between the three phages included 9 variant genes located in two regions of small, primarily hypothetical novel proteins in the left arm (data not shown). Notable similarities were found between the genomes and morphologies of these three phages and two previously described phages, *Rhodococcus equi* phage ReqiDocB7 (GenBank NC_023706) and *Gordonia* phage GTE7 (GenBank NC_016166) (Figs 2 and 3)^31^,^33^. The similarity between the three new *Gordonia* phages, ReqiDocB7 and GTE7 was based on overall morphological characteristics, most notably the long, flexible tails with lengths of 438–520 nm, and genome arrangement, rather than nucleotide sequence. Out of 98 unique proteins, phages GordTnk2, Gmala1, and GordDuk1 shared 28 with DocB7, and 26 with GTE7, albeit all at less than 51% identity (Fig. 3A). One of the more unusual features present in all five phage genomes is a long region upstream of the right terminus with multiple inverted repeats and no protein coding genes. This region is ~1,800 bp in GordTnk2, Gmala1, and GordDuk1, which is intermediate in length to the analogous region of GTE7 (1,185 bp) and ReqiDocB7 (2,484 bp). Such large stretches of non-coding sequence are unusual in phage genomes. Another shared feature of these phages is the complexity of the predicted lysis genes. Between genes 22 and 33 of GordTnk2, Gmala1, and GordDuk1, there are four genes (22, 24, 26 and 33) whose products are predicted to function in cell wall hydrolysis, and three genes encoding proteins with transmembrane domains (TMDs) (gp27, gp30, and gp31) that could function as membrane-permeabilizing holins (Fig. 4 and Table 3).

For the purposes of bacterial control, virulent phages (i.e., phages unable to form lysogens) are preferred^37^. Based on analysis of the genomes of phages GordTnk2, Gmala1, and GordDuk1, no evidence was found suggesting a temperate lifestyle in these phages. These phages were not found to be closely related to known temperate phages or to any prophage elements incorporated into sequenced bacterial genomes. A tyrosine recombinase was identified (gp72 in GordTnk2), and such recombinases can function as phage integrases. However the small size of gp72 and its relationship to bacterial XerC-like recombinases suggests this protein is more likely to function as a general recombinase during phage DNA replication. Efforts to isolate lysogens of phages GordTnk2, Gmala1, and GordDuk1 were unsuccessful (data not shown). Taken together, the data support a model in which phages GordTnk2, Gmala1 and GordDuk1 are virulent phage not capable of lysogeny.

### Genome of Gsput1

The 43,505 bp genome of Gsput1 was also determined and predicted to encode 71 proteins (Table 2, Fig. 3B). Gsput1 was found to be unrelated to phages GordTnk2, Gmala1, and GordDuk1 at both the DNA and protein levels. The Gsput1 genomic DNA was determined to possess 16 bp 5′ extended *cos* termini with the sequence 5′-GCTGACCCACGGACAC-3′. There was no DNA-level similarity between Gsput1 and any phage in the public database. Of 71 proteins, 41 proteins were found to share sequence similarities to previously identified proteins, primarily those encoded by mycobacteriophage. However, there were no extensive similarities to any single organism; the most closely related phage genome was that of mycophage Redi (NC_023730, eight similar proteins), and the most closely related prophage elements are located in *Nocardia farcinica* (NC_006361, 15 similar proteins across three genomic loci) and *Corynebacterium diptheriae* (BX248353, 12 proteins across three loci). To date, some 891 mycophage genomes have been determined and organized into 21 clusters and nine singletons based on nucleotide similarity (http://phagesdb.org/clusters/)^30^,^38^,^39^. Individual Gsput1-encoded proteins were similar to proteins distributed among 27 of the 30 mycobacteriophage clusters/singletons (Fig. 3B). Gsput1 is probably temperate, as it encodes a predicted integrase (gp28) which shares strong homology with integrase proteins found in the genomes of other *Mycolata* (E < 10^−100^) and with prophage integrases (e.g., *Lactobacillus* prophage Lj965 E = 10^−17^). Additionally, Gpsut1 gp29 encoded directly upstream of gp28, contains a detectable excisionase (Xis) domain (TIGR01764), further supporting the prediction of a functional Int/Xis module and temperate lifestyle.

### Phage efficacy test in a model activated sludge system

**Figure 5:**
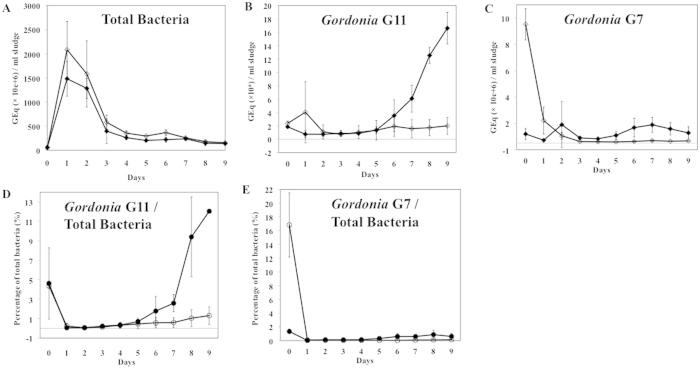
Effect of phage on total bacterial and *Gordonia* levels in laboratory-scale activated sludge systems. *Gordonia* strains G11 and G7 were inoculated to activated sludge with (empty mark) or without (filled mark) phage cocktail treatment. Quantities (expressed as genome equivalents, or GEq per ml sludge) of total bacterial (**A**), *Gordonia* G11 (**B**), *Gordonia* G7 (**C**), percentage of total bacteria of *Gordonia* G11 (**D**) and G7 (**E**) were determined. Of each treatment (with or without phage), the mean values of two parallel sludge systems were plotted, with the error bars indicating the standard deviations.

Preliminary standard phage host killing assays in defined liquid media demonstrated that treatment of pure *Gordonia* cultures with phages controlled host cell growth (data not shown). The capacity of the four phages to affect *Gordonia* accumulation in the presence of the mixed chemistry and microbiology of activated sludge was examined. Activated sludge was collected from the local wastewater treatment plant and split into separate aerated bioreactors to start the experiment immediately. Parallel bioreactors for each treatment (control or phage treated) were set up in order to conduct the experiment in duplicate. The sludge was inoculated with *Gordonia* hosts, and phage treatments were added two hours after the host inoculation. Dynamics of total bacteria and *Gordonia* accumulation, expressed as a function of genome equivalents (GEq) per ml sludge, as well as the relative abundance of *Gordonia* as compared to total bacteria in the bioreactors, was monitored over a nine-day period by RT-qPCR. The level of total bacteria in the fresh sludge, prior to inoculation, was measured at 5.8 × 10^7^ GEq/ml sludge. *Gordonia* host background levels in the sludge were measured, and native *Gordonia* sp. G11-like organisms were not detected while *Gordonia* sp. G7-like organisms were determined to be present at 8 × 10^4^ GEq/ml sludge (data not shown). In both control and phage-treated systems after the start of the experiment, the total bacteria level increased by ~25 fold during day one, rising from 5.9 × 10^7^ to ~2 × 10^9^ GEq/ml sludge. Following this initial increase, total bacteria levels decreased between days one to three and reached a level around 2 × 10^8^ GEq/ml sludge by day three and onwards (Fig. 5A). The inoculated *Gordonia* host strains exhibited different dynamics than did total bacteria. Following inoculation into the mixed sludge bioreactors, the host *Gordonia* strains exhibited an initial fluctuation in levels, primarily during the first day. By day two, both hosts stabilized and thus allowed observation of phage effect on *Gordonia* levels over the nine-day course of the experiment (Fig. 5B–E). In the phage treated systems, both G7 and G11 levels remained essentially constant after day two, however significant growth of G7 and G11 with different dynamics was observed in the non-phage treated systems. *Gordonia* G11 levels at the end of nine days were on average 1.6 × 10^7^ GEq/ml sludge, or 12.0% of total bacteria. This is over 10-fold higher than G11 levels in phage-treated reactors, at 4.0 × 10^6^ GEq/ml sludge, or 0.7% of total bacteria (Fig. 5B,D). In contrast, host G7 did not grow as well in the bioreactors as did G11, with the highest cell levels observed at day seven, which was followed by a steady decrease. Because of the reduced growth of G7, even though phage-treated samples showed little increase in host G7 level, on both GEq/ml and percentage of total population basis, the difference between phage treatment and control was less pronounced. Despite this, the G7 level in untreated sludge at day nine of the experiment (7.8 × 10^5^ GEq/ml sludge, or 0.6% of total bacteria) was still higher than the G7 level in the phage treated sludge (<2.0 × 10^5^ GEq/ml sludge, or <0.1% of total bacteria) (Fig. 5C,E).

Variations in bacterial cell growth in two parallel sludge systems were very high, resulting in broad standard deviations at each time point (Fig. 5). This is not unexpected given the heterogeneous nature of the complex microbial populations in sludge matrix. An independent duplication of the experiment was conducted using a different batch of activated sludge as the matrix. Despite the inherent variability that arises when utilizing such a mixed test matrix, phage treatment suppressed *Gordonia* host levels compared to non-treated samples (Supplementary Fig. S2).

**Figure 6:**
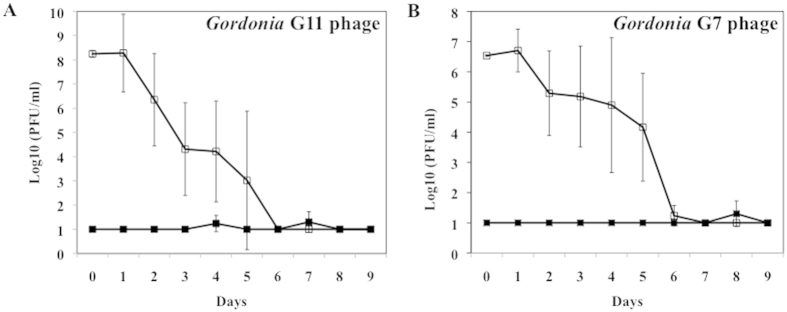
Recoverability of phage against *Gordonia* in the laboratory-scale activated sludge systems. *Gordonia* strains G11 and G7 were inoculated to activated sludge with (empty mark) or without (filled mark) phage cocktail treatment. Mean levels of phage against host G11 (**A**), and host G7 (**B**), in two parallel sludge systems were plotted with error bars indicating standard deviations.

The level of recoverable phage in the sludge systems was also assayed at each time point by pfu-based titration of both phage-treated and untreated samples, on lawns of host G11 and G7 (Fig. 6). Phage was undetected (<10 pfu/ml, 10 pfu/ml is the detection limit) in sludge samples inoculated with *Gordonia* hosts G11 and G7 without phage treatment. While the phage-treated sludge samples initially had high levels of phage, phage titers showed consistent declines over the course of the experiments. Though a decrease in recovered phage titers was observed over time, phages against G11 and G7 could still be recovered (≥10 pfu/ml) up to six days after initial inoculation (Fig. 6A,B, respectively).

Finally, the morphology and settling property of sludge floc were compared between the freshly collected sludge at the start of the experiment (day zero), as well as phage-treated and untreated control sludge at the end of the experiment (day nine) (Table 4). At the end of phage treatment, both control and phage-treated sludge had significantly reduced amount of filaments around flocs compared to the freshly collected sludge. The freshly collected sludge at the start of the experiment and the phage-treated sludge at the end of the experiment had SVI values of 75.3 and 97.3 ml/g, respectively. These SVI values were higher than the SVI value of the untreated control sludge at the end of the experiment (54.9 ml/g). After sludge settling, the supernatants above the solids layer were collected and tested for foaming potential using the Alka-Seltzer® foaming test. The supernatant of the control sludge without phage treatment exhibited a higher foaming potential, evident by the greater maximum foaming volume and longer foam collapse time, compared to the phage-treated sludge and fresh sludge (Table 4). This is possibly caused by the high turbidity of the supernatant due to poor sludge settling of untreated sludge.

## Discussion

Foaming is a common and ubiquitous problem in sludge management resulting in elevated costs and reduced operation efficiencies. Foaming is frequently the result of overgrowth of nocardioform filamentous bacteria, including *Nocardia*, *Gordonia*, *Rhodococcus*, and *Corynebacterium* spp. Though currently unidentified factors could determine the foaming process, there is evidence that *Gordonia* is commonly the predominant genus within the foaming-associated mycolata^15^,^18^,^19^. Current control methods include mechanical removal of foam, the use of chemical defoamers such as silicone sprays and polypropylene glycols, and implementation of biological control through supporting the growth of non-foaming bacteria. An improved foaming control methodology must be easily incorporated into current waste treatment protocols, be non-toxic, and affect only the target microorganisms while leaving non-target species intact. These control criteria might be met by the use of phage. Phages are already a normal constituent of the wastewater microcosm and are generally host–specific. Phage application does not usually completely eliminate the target host, which is advantageous as the target filamentous bacteria at a lower level are a necessary component in promoting floc-forming and proper sludge settling during activated sludge treatment process^2^. There are, however, challenges in the development of phage-based foaming control agents. Propagation of the necessary phage isolates may be problematic because some of the target filamentous bacteria associated with sludge foaming have been reported to be unculturable or extremely difficult to grow in laboratory settings^5^,^6^,^7^. Additionally, the full extent of diversity of bacteria responsible for the foaming remain to be discovered^15^. Finally, phage preparations have to be shown to be effective in the mixed chemistries and microflora of activated sludge, rather than just in defined culture media.

The approach used here for isolating foaming-associated filamentous bacteria was to culture activated wastewater sludge on minimal media with defined carbon sources, in order to reduce the growth of rapidly dividing bacteria and allow microcolonies of filamentous bacteria to form. Sodium acetate and glucose generally were better for growth of filamentous bacteria, compared to other carbon sources tested. Isolating and culturing different morphotypes of foaming-associated filamentous bacteria from wastewater foams is challenging and the currently acceptable identification of filamentous bacteria was thus mainly conducted *in situ*, using methods based on their *in situ* morphological features and/or molecular FISH method using 16s rRNA-based DNA probes^16^. These results suggest a feasible approach for isolating *Gordonia* and/or other filamentous bacteria from wastewater sludge samples.

Using the isolated *Gordonia* strains as hosts, phages isolated from wastewater were able to control *Gordonia* levels in defined media (data not shown). Moreover, application of these phages resulted in repeatable, significant suppression of *Gordonia* levels in activated sludge conditions. This is surprising, given the considerable diversity and species richness observed in the micro- and macro-organism community of the activated sludge, with different organismal groups responsible for complex functions including floc-forming, phosphorus removal, nitrite oxidation, and denitrification^3^,^7^. In such a heterogeneous and complex background consisting of viruses, bacteria, protozoa and metazoans, experiment-to-experiment variations are to be expected. Following immediate inoculation into the mixed sludge bioreactors, the host *Gordonia* values fluctuated at the beginning of the experiment (day zero to day one) (Fig. 5), possibly reflecting the stage required for the exogenously added *Gordonia* to be equilibrated into the sludge microbial system. Upon *Gordonia* and phage inoculation (day zero), a high level of *Gordonia* G7 was observed in phage-treated system compared to the control. This possibly reflects the rapid initial phage lysis and release of G7 genomic DNA, which resulted in efficient *Gordonia* DNA extraction and thus increased detectable quantities of *Gordonia* 16S rRNA genes. Despite the fluctuations at the start of the experiment, the effect of phage-treatment on *Gordonia* populations in the activated sludge was reproducibly detectable between day five and day nine. In these experiments, the level of indigenous background *Gordonia* sp. G11-like organisms was below the detection limit. In addition, there was very low level of indigenous *Gordonia* sp. G7-like organisms in the fresh sludge. The phage applied to the sludge system may have had a killing effect on both the indigenous and the exogenously inoculated *Gordonia* hosts. This study provides information on phage-*Gordonia* population dynamics in real activated sludge on a laboratory scale, conditions that more closely mimic the real world treatment systems compared to efficacy studies carried out in defined cultures^33^,^36^.

In addition to controlling *Gordonia* levels in activated sludge, phage-treated sludge at the end of the experiment showed better settling properties compared to the control. Compared to the continuous-flow aeration reactors, which resemble continuous biological enrichment cultures, the activated sludge aeration systems in this study were aerated static cultures. Compared to the freshly collected sludge, both the control and phage-treated sludge systems at the end of the experiment showed only small amount of filaments around less compact flocs. This suggested the poor health of flocs after prolonged aeration (nine days) at static culturing mode. Nevertheless, phage treated sludge had a sludge volume index (SVI) of 97.3 ml/g, which falls in the range of what is typically expected in a healthy sludge aeration basin (70–150 ml/g). SVI is an indicator best characterizing sludge settling properties. A SVI of greater than approximately 150 ml/g is often classified as bulking, while a SVI of less than approximately 70 ml/g can leave behind a turbid supernatant, the condition known as “pin-point floc”^40^. Compared to the control sludge with a low SVI (54.9 ml/g), the phage treated sludge at the end of the experiment had lower foaming potential in its supernatant after settlement, indicating more complete sludge settlement.

In conclusion, this study has demonstrated that phages applied for bio-control can survive during sludge aeration. Moreover, phages exhibited the capability for suppressing *Gordonia* hosts in real sludge and improving sludge settling property. Taken together, these results indicate significant potential for phage application in controlling *Gordonia*-associated foaming and bulking during wastewater treatment.

## Materials and Methods

### Isolation and identification of filamentous bacteria from wastewater foams

Activated sludge foam samples were collected from a local municipal wastewater treatment plant. After dispersing the flocs using low energy sonication in an ultrasonic water bath, samples were diluted and plated on Brain Heart Infusion (BHI, Difco) agar, and minimal salt medium base (0.5 g/l (NH_4_)_2_SO_4_, 0.01 g/l Ca(NO_3_)_2_, 0.05 g/l K_2_HPO_4_, 0.05 g/l MgSO_4_·7H_2_O, 0.05 g/l KCl, 0.1 g/l CaCO_3_) supplemented with different carbon sources (0.15 g/l sodium acetate, sucrose, glucose, or sodium succinate). The plates were incubated at 30 °C and were examined daily under a phase contrast microscope at 100–400× magnification over one month for micro-colony development. Bacteria exhibiting filamentous growth were picked using a fine needle. These strains were purified and routinely maintained on BHI agar at 30 °C.

The identity of each isolate was determined by amplification of the 16S rDNA genes using the primer set 16S.F (5′-GGTGAGTAACACGTGGGTGA-3] and 16S.R (5′-GGGGTCGAGTTGCAGACC-3′) and sequencing the resulting ~1200 bp amplicon. The 16S rDNA sequences were analyzed via the Ribosomal Database Project (RDP) 10 suite (rdp.cme.msu.edu). The closest three matching type strain sequences for each isolate were determined by the RDP SeqMatch tool^41^. Sequences were aligned with ClustalX v2.1 and a neighbor joining tree was generated with gap positions excluded^42^. Strains were assigned at the species level based on >99.5% sequence identity to a known bacterial strain as determined from the sequence alignment. Strains with <99.5% identity or >99.5% identity to more than one species were assigned at the genus level only. The tree was visualized and rendered with Archaeopteryx v0.957^43^.

### Phage isolation and host range determination

Foam samples collected from a local wastewater plant were centrifuged (10,000 × g, 10 min), and the filtered supernatants (through 0.22 μm filters) were mixed 1:1 with 2× BHI for phage enrichment. Phage detection and preparation were performed using BHI as top agar following standard methods^44^. BHI (0.5×, ~pH 6.5) was routinely used as buffer to suspend phage. The host range of phages was assessed by spotting 10 μl of lysates at routine test dilutions (RTD) onto lawns of individual hosts. The RTD of phage was defined as the last 10-fold dilution that developed confluent clearing when spotted onto a lawn of the original enrichment host.

### TEM

Transmission electron microscopy (TEM) was performed by diluting lysates 1:1 to 4:1 with TEM buffer (20 mM NaCl, 10 mM Tris-HCl pH 7.5, 2 mM MgSO_4_) and applying to 10–15 nm carbon films by the Valentine method^45^. Images were generated as previous described^31^.

### Phage genome sequencing and annotation

Phages were sequenced to ~23-fold coverage by pyrosequencing (Roche/454 Life Sciences, Branford, CT) following the protocols described previously^46^. Gap closure was completed by sequencing PCR products generated by the amplification of gap regions. The genomic terminal structure and end sequences were determined based on terminase homology and genome circular assembly, or based on direct sequencing of the termini using phage genomic DNA as template, and self-ligating of phage genomic DNA followed by PCR and sequencing of the PCR product^46^. Protein-coding genes were initially predicted using Genemark.hmm^47^ and manually edited in Artemis^48^. Predicted proteins were searched against the GenBank nr database using BLASTp^49^. Conserved domains were detected with InterProScan version 4.7 run locally^50^. Protein localization was determined by analysis with TMHMM 2.0 (http://www.cbs.dtu.dk/services/TMHMM). Phage genome maps were rendered with DNA Master (http://cobamide2.bio.pitt.edu/computer.htm).

### Nucleotide sequence accession number

The 16S rDNA sequences of the *Gordonia* isolates, were deposited in the GenBank database under the accession numbers KR067658 (G1), KR067659 (G4), KR067660 (G5), KR067661 (G7), KR067662 (G8), KR067663 (G10), KR067664 (G11). Accession numbers for phage genome sequences are: KP790008 (GordTnk2), KP790009 (Gmala1), KP790010 (GordDuk1), KP790011 (Gsput1).

### Phage efficacy test in lab-scale activated sludge systems

Activated sludge freshly collected from a local wastewater plant (College Station, TX) was divided into four parallel one-liter Pyrex® ProCulture™ Spinner Flask bioreactors (4502-series, Corning) with central magnetically driven impeller assembly and two dual angled sidearm fittings. Continuous aeration in the form of filtered sterile air was introduced via longer tubes of both side arm fittings to the bottom of the bioreactors, and the head space air venting was achieved via the shorter tubes of the side arm fittings. The submerged air bubbling, coupled with magnetically driven stirring of the culture, was used to supply adequate aeration to the activated sludge. Each bioreactor contained 500 ml of activated sludge. A portion (~150 ml) of the clear water phase was removed, to compensate for the volume increase due to bacteria and phage inoculation.

The bioreactors were inoculated with host bacteria to a final level of 5 × 10^7^ cfu/ml. This was achieved by application of 25 ml of a 1 × 10^9^ cfu/ml fresh preparation of each strain (G7 and G11). Two hours after *Gordonia* inoculation, 100 ml phage cocktail was applied to two of the four bioreactors. The final concentration of phage in the phage treated bioreactors was 6.5 × 10^8^, 9.5 × 10^8^, 3.7 × 10^8^, and 1.0 × 10^9^ pfu/ml for phages Gsput1, GordTnk2, Gmala1, GordDuk1, respectively. This corresponded to approximate phage treatment MOI of each phage of 5–15. Blank phage buffer (0.5× BHI, 100 ml) was inoculated into the other two bioreactors to serve as non-phage controls. Fresh activated sludge alone (without *Gordonia* or phage inoculation) was tested in parallel. At the start of the experiment and each day after (up to nine days), aliquots (1.5 ml each) of well-mixed sludge samples were taken aseptically using a syringe fitted through sidearm fittings, and were used for phage titering and DNA isolation. The experiment was carried out independently twice, each time using freshly collected sludge. At the end of each of experiment, sludge testing was conducted as described below.

### Quantification of *Gordonia* and total bacteria in activated sludge experiments using real time PCR

Total DNA was isolated from sludge using the Mo Bio UltraClean™ Microbial DNA Isolation kit (Mo Bio Laboratories, CA) utilizing bead-beating step. For *Gordonia* strains G7 and G11 quantification, the following primers and TaqMan probes targeting specific 16sRNA regions were designed in this study: *Gordonia* sp. strain G7, G7.F (5′- CTGGGAAACTGGGTCTAATAC); G7.R (5′- CATCCCAAACCGCAAAAG); G7.P (5′-TTCCACCACAAGACATGCATCCTGA); *Gordonia* sp. strain G11, G11.F (5′-CTGGGAAACTGGGTCTAATAC); G11.R (5′- CATCCCTAACCGCAAAAG); G11.P (5′- TCCACAAATCCCCATGCGAGGAA). For quantification of total bacteria, 16sRNA primers/probes reported previously were used: BacT.F (5′-TCCTACGGGAGGCAGCAGT); BacT.R (5′-GGACTACCAGGGTATCTAATCCTGTT), BacT.P (5′- CGTATTACCGCGGCTGCTGGCAC)^51^. PCR were performed in 384-well plates using a 7900HT Fast Real-Time PCR System (Applied Biosystems, Foster City, CA) and the results were analyzed using SDS v2.3 (Applied Biosystems). For validating and calibrating the G7 and G11 primer/probe sets, standard curves were generated in the range of 0.025−50,000 pg total DNA per reaction, using G7 and G11 genomic DNA, respectively. For calibrating total bacteria primer/probe set, G11 genomic DNA was used to generate standard curve. Quantification efficiencies, detection range, and Ct were calculated from the standard curves. The optimal sludge DNA input level was determined to be 10 ng based on the Ct values for G7, G11, and total bacteria quantification in the DNA input range of 1–100 ng. To test real samples, Each 20-μl reaction mixture contained 10 μl of 2× TaqMan Universal Master mix II with UNG (Applied Biosystems), 10 ng total DNA, 625 nM each of forward and reverse primers (Integrated DNA Technologies, Coralville, IA), and 250 nM of probe (5′-/56-FAM/-/ZEN/-/3IABkFQ/-3′ probe, Integrated DNA Technologies). The thermal profile consisted of 50 °C for 2 min, 95 °C for 10 min, and 40 cycles of 95 °C for 15 sec, 60 °C for 60 sec. All samples were measured in duplicate in each assay. Standard curves (generated using G11 or G7 genomic DNA) and negative controls were included in each PCR run. The average size of the Gordonia genome was estimated to be 5.2 Mbp, based on the sequenced genome of *G. bronchialis* DSM43247, NC_013441). By comparison of the fluorescence generated by each activated sludge sample with standard curves, the quantities of bacterial and *Gordonia* 16s rRNA genes of activated sludge samples were interpreted as *Gordonia* genome equivalents per 10 ng of extracted DNA used in PCR reaction. This value was then adjusted for the total DNA extracted from 1.5 ml sludge, and the final result was expressed as *Gordonia* genome equivalents per ml of sludge.

### Activated sludge testing

Floc morphology was examined using a phase-contrast microscope (Fisher Scientific Micromaster^TM^, Pittsburgh, PA) under 1000× magnification. The total suspended solids concentration of sludge was determined by filtering and drying sludge aliquots following the standard method^52^. The sludge volume index (SVI) was determined by transferring the sludge to a 1 liter graduated cylinder, and allowing the sludge to settle undisturbed for 30 min. The volume occupied by the settled sludge was recorded and used to calculate the SVI (ml/g) = settled sludge volume (ml/l)/total suspended solids (g/l). After sludge settlement, the supernatant above the settled layer was subjected to the Alka-Seltzer® foaming potential test as described previously^1^.

## Additional Information

**How to cite this article**: Liu, M. *et al.* Bacteriophages of wastewater foaming-associated filamentous *Gordonia* reduce host levels in raw activated sludge. *Sci. Rep.* **5**, 13754; doi: 10.1038/srep13754 (2015).

## Supplementary Material

Supplementary Information

## Figures and Tables

**Table 1:** Host range of 14 Gordonia phages determined as determined by spotting at the routine test dilution.

Bacterial strain	Bacteriophage isolate													
	Group 1		Group 2			Group 3					Group 4			
	Gordnk1	GordTnk2	Gmala1	Gmala2	Gmala3	GordDuk1	GordDuk2	GordDuk3	GordDuk4	GordDuk5	Gsput1	Gsput2	Gsput3	Gsput4
*Gordonia* sp. G1	+	+												
*G. malaquae* G4			+	+	+									
*Gordonia* sp. G7	+	+	+	+	+	+	+	+	+	+				
*G. sputi* G11											+	+	+	+

Note: “+” indicates clearing on the indicated host.

**Table 2:** *Gordonia* phage characteristics and genome feature summary.

Phage isolate	Virion headdiam (nm)	Tail length(nm)	Genomelength (bp)	GC%	Genometermini	No. of CDS
GordTnk2	74 ± 1	519 ± 6	75,987	50.7%	*pac*	98
Gmala1	72 ± 1	520 ± 6	75,167	50.8%	*pac*	90
GordDuk1	75 ± 3	520 ± 10	76,276	50.7%	*pac*	97
Gsput1	62 ± 1	229 ± 5	43,505	62.8%	5′ *cos*	71

**Table 3:** Lysis proteins in genomes of GordTnk2, Gmala1 and GordDuk1.

Protein	Predicted Lysis Function	Features	Most Similar Proteins	Comments
gp22	cell wall degradation	N-terminal glycoside hydrolase domain (IPR013781)	*M. thermoresistibile* (WP_003925585), *M. iranicum* (WP_024446457), *M. gilvum* (WP_011891504)	Many mycobacteria homologues, but no phage homologues, no TAT signal sequence
gp24	LysA, cell wall degradation	N-acetylmuramoyl-L-alanine amidase domain (IPR002502)	*Gordonia* phage GTE7 gp28 (YP_004934729), *Streptomyces* phage Jay2Jay (AIW02547), *Mycobacterium* phage Jolie2 (YP_009009684) and *Rhodococcus* phage ReqiPepy6 (YP_009017624)	Similar to numerous phage encoded LysA equivalents
gp26	LysA, cell wall degradation	no conserved domain (truncated)	Rhodococcus phage ReqiDocB7 gp27 (YP_009013817), *Gordonia* phage GTE7 gp29 (YP_004934730)	truncated, Homologue is only LysA candidate in ReqiDocB7
gp27	holin, membrane disruption	4 transmembrane domain	*Gordonia* phage GTE7 gp26 (YP_004934727), *Mycobacterium* phage Omnicron holin (AIM50365), many mycophage holins	possible dual start
gp30	holin, membrane disruption	4 transmembrane domain	*Gordonia* phage GRU1 gp39 (YP_004935862), +3 other phage	possible dual start
gp31	holin, membrane disruption	1 transmembrane domain	*Gordonia* phage GRU1 gp40 (YP_004935863), +4 other phage	no dual start
gp33	LysB, mycolic acid hydrolyses	PE-PPE domain (IPR013228)	*Mycobacterium* phage Dumbo lysin B (YP_008051660)	not similar to GTE7 or DocB7 LysB

**Table 4:** Sludge characteristics before and after phage treatment.

Characteristics	Start of experiment (Day 0)	End of experiment (Day 9)	
	Fresh blank sludge	No phage control	Phage treated
Floc morphology^a^	significant amount of short filaments around compact flocs	slight amount of short filaments, less compact flocs	very few visible filaments, less compact flocs
Total suspended solids (g/l)	1.86	2.35	3.7
Sludge volume index (SVI) (ml/g)	75.3	54.9	97.3
Foaming test (max. volume/collapse time)^b^	120 ml/4 min 39 s	170 ml/8 min 20 s	130 ml/5 min 10 s

^a^Examined under phase contrast microscope at 1000×.

^b^Tested using the liquid portion after sludge settling for 1 h, and max foam volume and callapse time to see 50% clear surface were recorded.
