# Supplementary material for: Bacteriophages of wastewater foaming-associated filamentous Gordonia reduce host levels in raw activated sludge
Source: Sci Rep. 2015 Sep 9;5:13754. doi: 10.1038/srep13754 (PMC4563357; doi:10.1038/srep13754)
Supplement: Supplementary Information [file srep13754-s1.pdf]

Bacteriophages of wastewater foaming-associated filamentous *Gordonia* reduce host levels in raw activated sludge

Mei Liu [a], Jason J. Gill [b,c], Ry Young [b,d], Elizabeth J. Summer [a]*

[a]Ecolyse Inc., 11142 Hopes Creek Rd., College Station, Texas 77845, USA; [b]Center for Phage Technology, 2128 TAMU, Texas A&M University, College Station, TX 77843, USA; [c]Department of Animal Science, 2471 TAMU, Texas A&M University, College Station, TX 77843, USA; [d]Department of Biochemistry and Biophysics, 2128 TAMU, Texas A&M University, College Station, TX 77843, USA

*Address correspondence to Elizabeth J Summer, liz@ecolyse.com

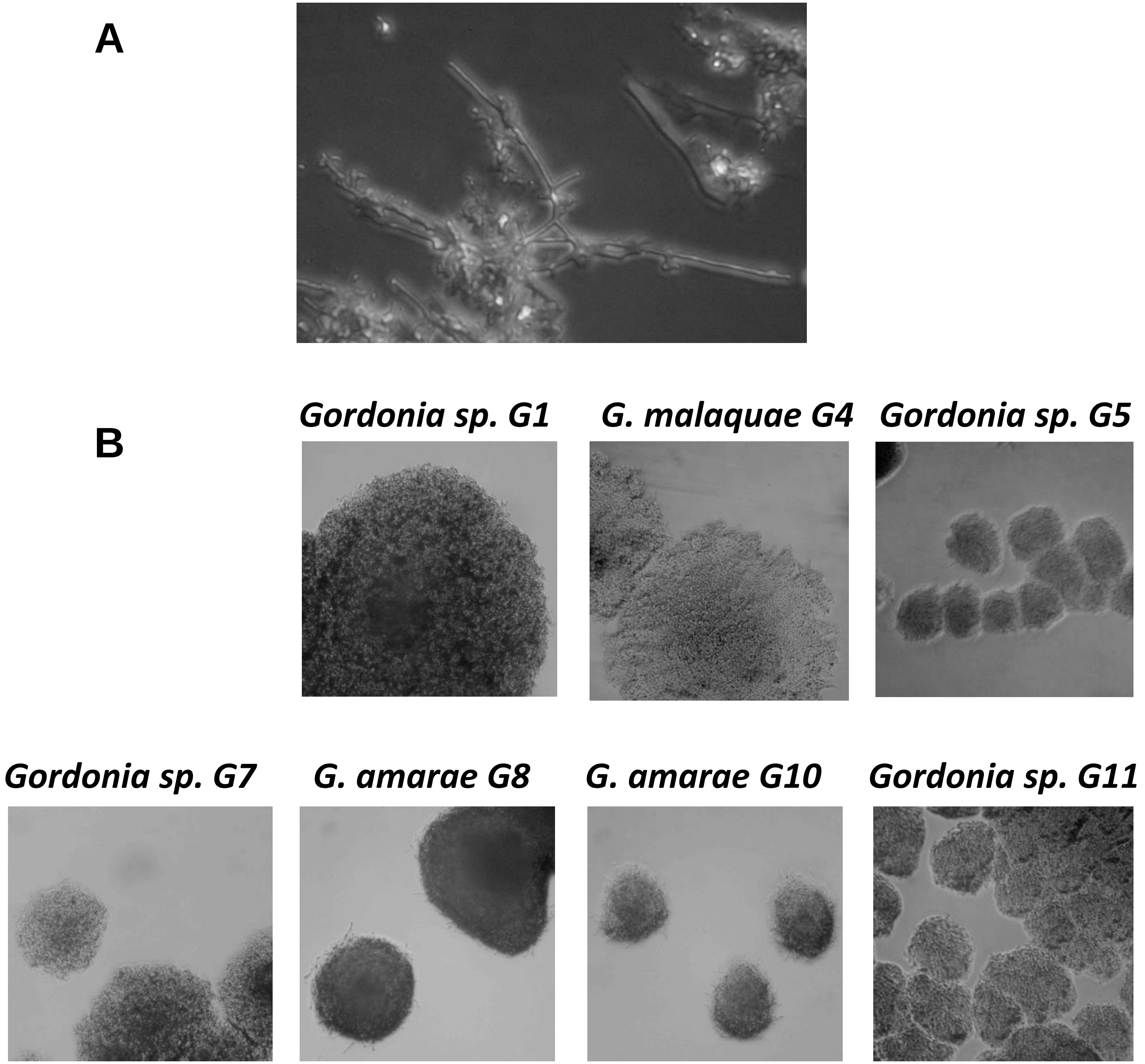

Supplementary Figure S1. Isolation of filamentous bacteria from wastewater foam samples. (A). Foam sample showing branched filamentous bacteria under phase contrast microscope (1,000 × magnification). (B). Micro-colony morphology of the *Gordonia* isolates, *Gordonia* sp. G1, *G. malaquae* G4, *Gordonia* sp. G5, *Gordonia* sp. G7, *G. amarae* G8, *G. amarae* G10, *Gordonia* sp. G11, examined under phase contrast microscopy (100 × magnification).

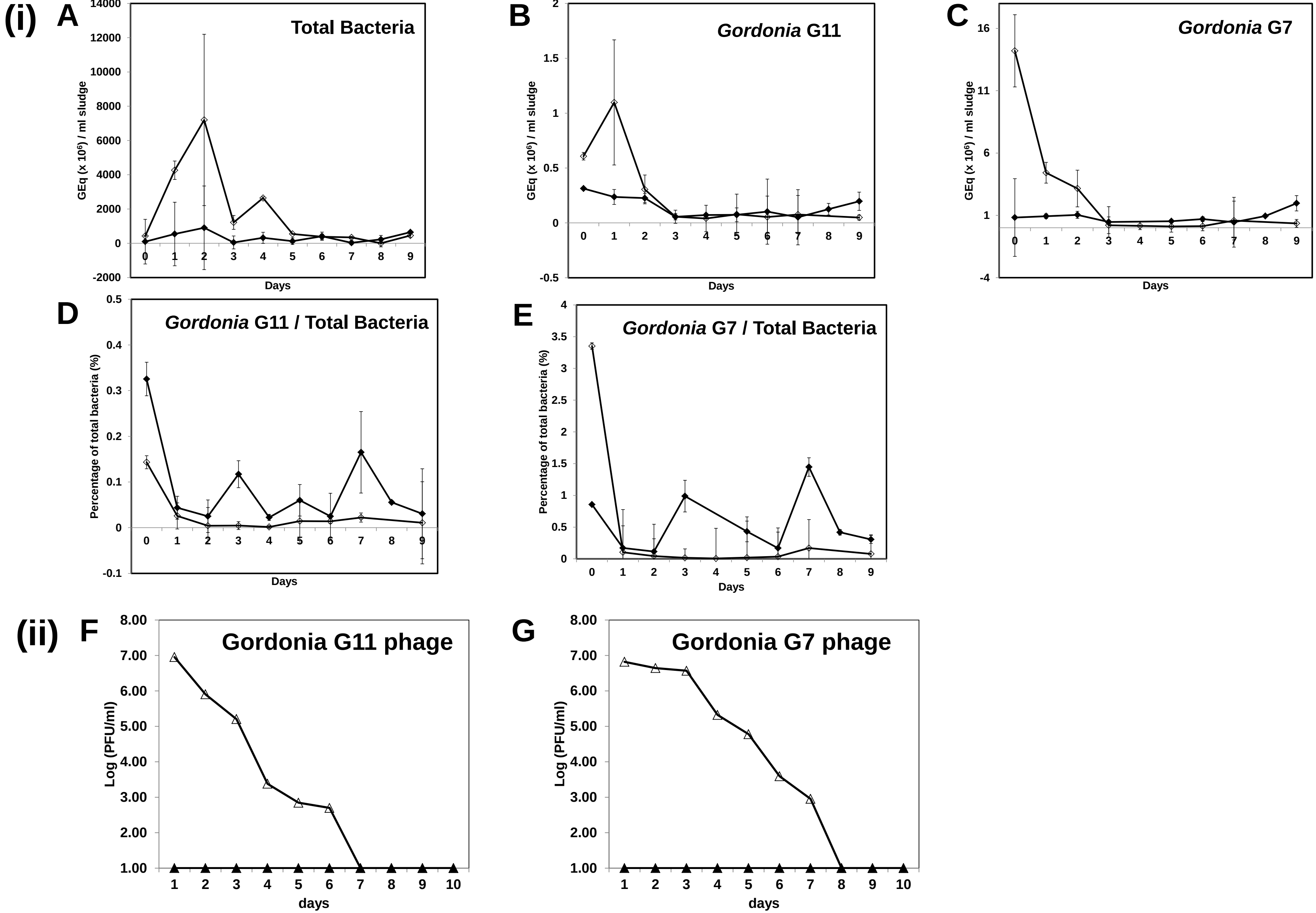

Supplementary Figure S2. Effect of phage on total bacterial and *Gordonia* levels in laboratory-scale activated sludge systems in an independently repeated experiment. *Gordonia* strains G11 and G7 were inoculated to activated sludge with (empty mark) or without (filled mark) phage cocktail treatment. (i) Quantities (expressed as genome equivalents, or GEq per ml sludge) of total bacterial (A), *Gordonia* G11 (B), *Gordonia* G7 (C), percentage of total bacteria of *Gordonia* G11 (D) and G7 (E). (ii) Levels of phage against host G11 (A), and host G7 (B). The error bars indicate the standard deviations of two duplicate experiments.

Supplementary Table S1. Predicted proteins, gene starts and annotations of phage GordTnk2.

Protein name	Start	Stop	Strand	Upstream Sequence	S-D	Start Codon	Predicted product	Evidence	TMHMM
gp01	393	2012	+	ctggcactgtgctagcgt GGT ccac	GGT	atg	hypothetical conserved protein		
gp02	2013	5048	+	ttccgatgttg AGGA agtgttctaa	AGGA	atg	TerL	IPR028992	
gp03	5183	7054	+	tgtcccgaacaca AGGAG aaatgta	AGGAG	atg	DUF935 like protein	IPR009279	
gp04	7110	7328	+	cctggggatatat GGA ctagaccat	GGA	atg	hypothetical novel protein		
gp05	7359	8525	+	cagacaatcagta AGGT ggttaacc	AGGT	atg	hypothetical conserved protein		
gp06	8540	9004	+	ttactctgtaag GGAG aaatagaaa	GGAG	atg	hypothetical conserved protein		
gp07	9017	10207	+	tacctttaagta AGGAG cctagaaa	AGGAG	atg	major capsid protein	IPR005564	
gp08	10240	10530	+	ttaaaccaga AGGA agtgaaatatc	AGGA	atg	hypothetical novel protein		
gp09	10652	11185	+	aattcgagcggaaa GGGG cgggcga	GGGG	atg	hypothetical conserved protein		
gp10	11182	11670	+	cgcccgctccgcc GGAGGT tattcc	GGAGGT	gtg	phage morphogensis protein	IPR006522	
gp11	11667	12170	+	aaacgtctattttt AGGA agctaaa	AGGA	gtg	hypothetical conserved protein		
gp12	12186	12491	+	gtttgagtaggaa AGGA acgagcaa	AGGA	atg	hypothetical novel protein		
gp13	12503	13528	+	ctgacgagaacta AGGAG taataaa	AGGAG	atg	hypothetical conserved protein		
gp14	13675	14280	+	catcaagcctagaa AGG gcaataag	AGG	atg	pre-TMP frameshift protein		
gp15	13675	14540	+	catcaagcctagaa AGG gcaataag	AGG	atg	pre-TMP frameshift protein		
gp16	14647	24465	+	caaccttca AGGA tattgagtccat	AGGA	atg	tape measure protein	IPR010090	
gp17	24468	27047	+	ttcttggactggc AGGAG cttagaa	AGGAG	atg	hypothetical conserved protein		
gp18	27067	28800	+	atttaagtagagaaa GAG aagggca	GAG	atg	hypothetical conserved protein		
gp19	28797	30644	+	gaaacggaaagc GAG ttactaagct	GAG	atg	hypothetical conserved protein		
gp20	30648	31685	+	gtggtctagcgaaac GGA atgagtc	GGA	atg	hypothetical novel protein		
gp21	31707	32090	+	ctagatttaatca AGGAG aaataaa	AGGAG	atg	hypothetical conserved protein		
gp22	32167	32868	+	catgaactaacgat AGGA tgagcgc	AGGA	atg	lysin	IPR013781	gp22 and gp24 both contain peptidoglycan-degrading domains, see text
gp23	32879	33244	+	ggaactctcgatta AGGAG aatgct	AGGAG	atg	hypothetical novel protein		
gp24	33244	33900	+	atatgctcggaa AGGT tgtaggcta	AGGT	atg	LysA	IPR002502	
gp25	33903	34169	+	agcttatgggaac GAGG gactaagg	GAGG	gtg	hypothetical novel protein		
gp26	34174	34542	+	gaaaacaccga AGGAG aataagatc	AGGAG	atg	LysA (truncated)		
gp27	34553	34999	+	agcaagattaagt GAGG aaatgttt	GAGG	atg	holin		4 tmd
gp28	34999	35538	+	atcctgacaag GGA atcaccggata	GGA	atg	methyltransferase	IPR029063	
gp29	35663	35872	+	agaacgctgggaatc GGT aacgggc	GGT	atg	hypothetical conserved protein		
gp30	35869	36360	+	atgaccattgcctt GAGG attggat	GAGG	ttg	holin		4 tmd
gp31	36363	36761	+	ttaaacaggaaga AGG cgagtaaga	AGG	atg	holin		1 tmd
gp32	36804	37133	+	ccatcagctactc AGGAG agagcaa	AGGAG	atg	Hypothetical novel protein		
gp33	37130	37915	+	agcaagcagga AGGT tcattttcac	AGGT	atg	LysB	IPR013228, PF08237,Hhpred: 1q66_A Acetyl xylan esterase I, 100.0; 4psc_A Carbohydrate esterase 100.0	
gp34	38018	38371	-	cgaatttacatgta AGGAG tgagaa	AGGAG	atg	hypothetical novel protein		
gp35	38375	38530	-	tgatttcccggacta AGGA tcataa	AGGA	atg	hypothetical novel protein		
gp36	38540	38824	-	tataagcac GGA cgaaagttctgaa	GGA	atg	hypothetical novel protein		
gp37	38826	39365	-	cggaaagaatctttaatt GGG gcga	GGG	atg	hypothetical conserved protein		
gp38	39329	39655	-	ctcaggctcggtat GGT ctagagat	GGT	atg	hypothetical novel protein		
gp39	39702	40127	-	gcattcaaga AGGA ttcaaaagtag	AGGA	atg	hypothetical conserved protein		
gp40	40120	40302	-	GAG taaataagaaagcggccaaagt	GAG	atg	hypothetical novel protein		2 tmd
gp41	40299	40661	-	ttctcatttttca AGGA cccgaaga	AGGA	gtg	hypothetical novel protein		
gp42	40609	41436	-	ttaagcaga AGG gccagtaataatt	AGG	atg	thymidylate synthase ThyX	IPR003669	
gp43	41442	42632	-	gattgatttgga GAGG ggaaatgaa	GAGG	gtg	hypothetical conserved protein		
gp44	42629	42811	-	accaaag AGGA ttggaactacgaac	AGGA	atg	hypothetical novel protein		
gp45	42884	43048	-	ctggcgtgaattaca GGGG gcagta	GGGG	atg	hypothetical novel protein		
gp46	43048	43290	-	tcaaattca AGGAGG gcgagaatta	AGGAGG	atg	hypothetical novel protein		
gp47	43290	43475	-	tgggccgg GGAG acgagaataagta	GGAG	atg	hypothetical novel protein		
gp48	43468	43692	-	c GGT gattcttctaaattcttccaa	GGT	atg	transcription factor WhiB	IPR003482	
gp49	43758	44006	-	taacgagtggta AGGA ttttagata	AGGA	atg	hypothetical novel protein		
gp50	44019	44573	-	ttttgattacagtgatt GGT gcaat	GGT	atg	RusA-like ribonuclease	IPR008822	
gp51	44570	44821	-	gagatttgtggagaga AGGT gaatg	AGGT	atg	hypothetical novel protein		1 tmd
gp52	44799	45017	-	tgtcaatcaacatg GGAG ttaagta	GGAG	atg	hypothetical novel protein		
gp53	45017	45208	-	gggcaaa GAG aaagcgaactgggcg	GAG	atg	hypothetical novel protein		2 tmd
gp54	45205	45357	-	atgcaccaaatgcc GAG aaggaata	GAG	atg	hypothetical novel protein		
gp55	45357	45665	-	caggtagaaatcga AGGAG cataaa	AGGAG	atg	hypothetical novel protein		
gp56	45667	46803	-	gaaattggaatt GGAG taattagat	GGAG	atg	DNA polymerase	TIGR00663, cd00140	
gp57	46810	46932	-	cctgtttattgagt GAGG aatcgaa	GAGG	atg	hypothetical novel protein		
gp58	46942	47532	-	aagaaaccggagaa GAGG attaatc	GAGG	atg	ribonuclease	IPR012337	
gp59	47535	47792	-	aggaaaaagaaaa GAGG aataagaa	GAGG	atg	DNA binding protein	IPR011991	
gp60	47789	49330	-	tgaataagaa AGGAG cacggttaat	AGGAG	atg	helicase	IPR001650	
gp61	49349	49726	-	aagatttatcgagtagt GGA ggttc	GGA	atg	hypothetical novel protein		
gp62	49707	49940	-	cccaagcat GAGG acctgatctaaa	GAGG	atg	hypothetical novel protein		
gp63	49942	50397	-	aattcttcaagac AGGA tcaagaaa	AGGA	atg	DUF732 like protein	IPR007969	
gp64	50710	51375	-	aaaatcgaaaca AGGAG aaaacaaa	AGGAG	atg	hypothetical conserved protein		
gp65	51531	51821	-	cttaaacccagtagc GGAG tctaga	GGAG	gtg	hypothetical novel protein		
gp66	51823	52689	-	aagttcggaagattt GGAGG aactg	GGAGG	atg	hypothetical novel protein		
gp67	52619	53131	-	ctagtattttggaat GGT gagaaaa	GGT	atg	hypothetical conserved protein		
gp68	53124	53450	-	atcaactgtttggca AGGAG ctaaa	AGGAG	gtg	hypothetical novel protein		
gp69	53447	53731	-	tcaggtaattccggc GGT gaattaa	GGT	atg	hypothetical novel protein		
gp70	53732	54028	-	gaagatcaggaagagc AGGA aatca	AGGA	atg	hypothetical novel protein		
gp71	54021	54371	-	ggcgcacgaggc GGT gaatttctaa	GGT	atg	hypothetical novel protein		
gp72	54372	55169	-	cactaactcacagac AGGA aaagaa	AGGA	atg	integrase	IPR011010, IPR002104, IPR013762	
gp73	55306	55686	-	caaaataccgac AGGAG aacgaaaa	AGGAG	gtg	hypothetical conserved protein		
gp74	55683	56012	-	aaaatcaagagacag GGA tttatca	GGA	atg	hypothetical novel protein		
gp75	56142	57011	-	tagatatgatagaaac GGAG ggaaa	GGAG	atg	hypothetical novel protein		
gp76	57008	57394	-	tttattatgattaagt GGT cgggat	GGT	atg	hypothetical novel protein		
gp77	57521	58231	-	atcagagaaatgttct GGA acgata	GGA	atg	hypothetical novel protein		
gp78	58231	58473	-	gaccgaggaaagt GGGG agaaaaca	GGGG	gtg	hypothetical novel protein		
gp79	58470	58637	-	aaaagcacaggaac AGGT acaccct	AGGT	atg	hypothetical novel protein		
gp80	58634	58852	-	gaccatgcggaatt GGAG attgcaa	GGAG	atg	hypothetical novel protein		
gp81	58816	59262	-	t GGT tgacaaaagaaagaagtaatc	GGT	atg	Mycobacteriophage protein	IPR019627	
gp82	59265	59480	-	tccagcgatacgtcaagt AGG gaaa	AGG	atg	hypothetical novel protein		
gp83	59486	59926	-	cgaccaagaaagac GGT aaataaaa	GGT	atg	hypothetical novel protein		
gp84	59929	60687	-	acaaaatgccga AGGAG cgtgtcaa	AGGAG	gtg	methyltransferase	IPR029063	
gp85	60684	61304	-	cacagcacaaac AGGA aaataaaaa	AGGA	atg	hypothetical novel protein		
gp86	61431	61622	-	aatcaacgcatagaaagatttcacc	None	gtg	hypothetical novel protein		
gp87	62000	62182	-	ctgagattgaaaa GAGG attttgtg	GAGG	atg	hypothetical novel protein		
gp88	62182	64806	-	taatcaacacgagac AGGA aatctc	AGGA	atg	von Willebrand factor (vWF)	IPR002035	
gp89	64928	66745	-	tcactgtaaa GGGG attctgagaaa	GGGG	atg	ATPase	IPR027417	
gp90	67547	67792	-	caggcacggg GGA ttattgcgctct	GGA	gtg	hypothetical novel protein		
gp91	67948	68841	-	cggaatcacagac AGGA cagagaaa	AGGA	atg	hypothetical novel protein		
gp92	69229	69420	-	aactagcacagac AGGA ttaaagcc	AGGA	atg	hypothetical novel protein		1 tmd
gp93	69440	69694	-	aacactcacagac AGGA ctagaaca	AGGA	atg	hypothetical novel protein		
gp94	69819	70001	-	ccagcacagac AGGA cagaaataaa	AGGA	atg	hypothetical novel protein		
gp95	70115	70501	-	atcaacagagac AGGA caggacatt	AGGA	atg	hypothetical novel protein		
gp96	72310	74328	-	actcacagaacggcag GGA taatcg	GGA	gtg	DNA primase	IPR002694	
gp97	74332	74823	-	aaaaactagcgagaa AGGA cataac	AGGA	atg	hypothetical conserved protein		
gp98	74850	75608	-	cccggcgcggac AGGAG atataggt	AGGAG	atg	hypothetical conserved protein		

Supplementary Table S2. Predicted proteins, gene starts and annotations of phage Gmala1.

Protein name	Start	Stop	Strand	Upstream Sequence	S-D	Start Codon	Predicted product	Evidence	TMHMM
gp01	390	2021	+	ctggcactgtgctagcgt GGT ccac	GGT	atg	Hypothetical protein		
gp02	2022	5141	+	ttccgatgtt GAGG aagtgttctaa	GAGG	atg	TerL	IPR028992	
gp03	5276	7147	+	tgtcccgaacaca AGGAG aaatgta	AGGAG	atg	DUF935 like protein	IPR009279	
gp04	7203	7421	+	cctgggaatatat GGA ctagaccat	GGA	atg	Hypothetical novel protein		
gp05	7452	8618	+	cagacaatcagta AGGT ggttaacc	AGGT	atg	Hypothetical protein		
gp06	8633	9097	+	ttactctgtaag GGAG aaatagaaa	GGAG	atg	Hypothetical protein		
gp07	9110	10300	+	cacctttaagta AGGAG cctagaaa	AGGAG	atg	major capsid protein	IPR005564	
gp08	10333	10611	+	ttaaaccaga AGGA agtgaaatatc	AGGA	atg	Hypothetical novel protein		
gp09	10745	11278	+	aattcgagtggaaa GGGG cgggcga	GGGG	atg	Hypothetical protein		
gp10	11275	11763	+	cgccagctccgcc GGAGGT tattcc	GGAGGT	gtg	Phage morphogensis protein	IPR006522	
gp11	11760	12263	+	aaacgtctattttt AGGA agctaaa	AGGA	gtg	Hypothetical protein		
gp12	12279	12584	+	gtttgagtaggaa AGGA acgagcaa	AGGA	atg	Hypothetical novel protein		
gp13	12596	13621	+	ctgacgagaacta AGGAG taataaa	AGGAG	atg	Hypothetical protein		
gp14	13768	14373	+	catcaagcctagaa AGG gcaataag	AGG	atg	pre-TMP frameshift protein		
gp15	13768	14633	+	catcaagcctagaa AGG gcaataag	AGG	atg	pre-TMP frameshift protein		
gp16	14740	24558	+	caaccttca AGGA tattgagtccat	AGGA	atg	Tape measure protein	IPR010090	7 tmd
gp17	24561	27140	+	ttcttggactggc AGGAG cttagaa	AGGAG	atg	Hypothetical protein		
gp18	27160	28893	+	atttaagtagagaaa GAG aagggca	GAG	atg	Hypothetical protein		
gp19	28890	30737	+	gaaacggaaagc GAG ttactaagct	GAG	atg	Hypothetical protein		
gp20	30741	31778	+	gtggtctagcgaaac GGA atgagtc	GGA	atg	Hypothetical novel protein		
gp21	31800	32183	+	ctagatttaatca AGGAG aaataaa	AGGAG	atg	Hypothetical protein		
gp22	32260	32961	+	catgaactaacgat AGGA tgagcgc	AGGA	atg	lysin	IPR013781	see text
gp23	32972	33337	+	ggaactctcgatta AGGAG aatgct	AGGAG	atg	Hypothetical novel protein		
gp24	33337	33993	+	atatgctcggaa AGGT tgtaggata	AGGT	atg	LysA	IPR002502	see text
gp25	33996	34262	+	agcttatgggaac GAGG gactaagg	GAGG	gtg	Hypothetical novel protein		
gp26	34267	34635	+	gaaaacaccga AGGAG aataagatc	AGGAG	atg	LysA		
gp27	34646	35092	+	agcaagattaagt GAGG aaatgttt	GAGG	atg	holin		4 tmd
gp28	35092	35631	+	atcctgacaag GGA atcaccggata	GGA	atg	DNA methylase	IPR029063	
gp29	35756	35965	+	agaacgctgggaatc GGT aacgggc	GGT	atg	Hypothetical protein		
gp30	35962	36453	+	atgaccgttgcctt GAGG attggat	GAGG	ttg	holin		4 tmd
gp31	36456	36854	+	ttaaacaggaagaaggc GAG taaga	GAG	atg	holin		1 tmd
gp32	36897	37226	+	ccatcagctactc AGGAG agagtaa	AGGAG	atg	Hypothetical novel protein		
gp33	37223	38008	+	agcaagcagga AGGT tcattttcac	AGGT	atg	LysB	IPR029058, IPR013228, Hhpred: 1q66_A Acetyl xylan esterase I, 100.0; 4psc A Carbohydrate esterase 100.0	
gp34	38111	38464	-	cgaatttacatgta AGGAG tgagaa	AGGAG	atg	Hypothetical novel protein		
gp35	38468	38623	-	tgatttcccggacta AGGA tcataa	AGGA	atg	Hypothetical novel protein		
gp36	38633	38917	-	tataagcac GGA cgaaagttctgaa	GGA	atg	Hypothetical novel protein		
gp37	38919	39458	-	cggaaagaatctttaatt GGG gcga	GGG	atg	Hypothetical protein		
gp38	39422	39748	-	ctcagactcggtat GGT ctatggat	GGT	atg	Hypothetical novel protein		2 tmd
gp39	39949	40218	-	gcattcaagaa GGGT tcaaaagtag	GGGT	atg	Hypothetical novel protein		
gp40	40211	40393	-	gagtaaataaga AGG cggccaaagt	AGG	atg	Hypothetical novel protein		
gp41	40390	40752	-	ccgttatagtttttca AGGA cccgaaga	AGGA	atg	Hypothetical novel protein		
gp42	40772	41527	-	ttaagcaga AGG gccagtaataatt	AGG	atg	thymidylate synthase ThyX	IPR003669	
gp43	41533	42723	-	aattggtttgga GAGG ggaaatgaa	GAGG	gtg	Hypothetical protein		
gp44	42755	42916	-	ctggcgttaattata GGGG acagta	GGGG	atg	Hypothetical novel protein		
gp45	42916	43158	-	acgagaattagct AGGAG aaaagca	AGGAG	atg	Hypothetical novel protein		
gp46	43169	43387	-	c GGT gattctcccaaattcttccaa	GGT	atg	Transcription factor WhiB	IPR003482	
gp47	43453	43701	-	taacgagtggta AGGA ttttagata	AGGA	atg	Hypothetical novel protein		
gp48	43714	44268	-	ttttgattacagtgatt GGT gcaat	GGT	atg	RusA-like ribonuclease	IPR008822	
gp49	44265	44519	-	gaatagatttgtgaaaagaaggtga	None	atg	Hypothetical novel protein		1 tmd
gp50	44543	44695	-	atgcaccaaatgcc GAG aaggaata	GAG	atg	Hypothetical novel protein		
gp51	44695	45000	-	caggtagaaatcga AGGAG cataaa	AGGAG	atg	Hypothetical novel protein		
gp52	45002	46138	-	aagagttgaatt GGAG taattagat	GGAG	atg	Dna polymerase		
gp53	46224	46814	-	aagaaaccggagaa GAGG attaatc	GAGG	atg	ribonuclease	IPR012337, IPR013520, IPR006055	
gp54	46817	47074	-	aggaaaaagaaaa GAGG aataagaa	GAGG	atg	DNA binding protein	IPR011991, IPR009061, IPR010093	
gp55	47071	48612	-	tgaataagaa AGGAG cacggttaat	AGGAG	atg	Helicase	IPR014001, IPR001650, IPR027417	
gp56	48631	49002	-	tatcgagtagt GGAGGT tcacgaat	GGAGGT	atg	Hypothetical novel protein		
gp57	48989	49222	-	cccaagcatg AGGA cctgatctaaa	AGGA	atg	Hypothetical novel protein		
gp58	49224	49679	-	aattcctcaagac AGGA tcaagaaa	AGGA	atg	DUF732 like protein	IPR007969	
gp59	50000	50665	-	aaaatcgaaaca AGGAG aaaacaaa	AGGAG	atg	Hypothetical protein		
gp60	50821	51111	-	cttaaacccagtggc GGAG tctaga	GGAG	gtg	Hypothetical novel protein		
gp61	51113	51979	-	aagttcggaagattt GGAGG aactg	GGAGG	atg	Hypothetical novel protein		
gp62	51909	52421	-	ctagtattttggaat GGT gagaaaa	GGT	atg	Hypothetical protein		
gp63	52414	52740	-	atcaactattcggca AGGAG ctaaa	AGGAG	gtg	Hypothetical novel protein		
gp64	52737	53021	-	tcaggtaattccggt GGT gaattaa	GGT	atg	Hypothetical novel protein		
gp65	53022	53318	-	atcattcaggaagagc AGGA aatta	AGGA	atg	Hypothetical novel protein		
gp66	53311	53652	-	ggcgcacgaggc GGT gaatttctaa	GGT	atg	Hypothetical novel protein		
gp67	53653	54450	-	cactaactcacagac AGGA aaagaa	AGGA	atg	integrase	IPR011010, IPR002104, IPR013762	
gp68	54587	54967	-	caaaataccgac AGGAG aacgaaaa	AGGAG	gtg	Hypothetical protein		
gp69	54964	55293	-	aaaatcaagagacag GGA tttatca	GGA	atg	Hypothetical novel protein		
gp70	55423	56289	-	tagatatgatagaaac GGAG taaaa	GGAG	atg	Hypothetical novel protein		
gp71	56292	56675	-	tttattatgattaagt GGT cgggat	GGT	atg	Hypothetical novel protein		
gp72	56801	57511	-	atcagagaaatgttct GGA acgata	GGA	atg	Hypothetical novel protein		
gp73	57511	57753	-	gaccgaggaaagtag GGAG aaagca	GGAG	gtg	Hypothetical novel protein		
gp74	58096	58542	-	t GGT tgacaaaagaaagaaataatc	GGT	atg	Mycobacteriophage protein	IPR019627	
gp75	58545	58760	-	tcgagcgatacgtcaagt AGG gaaa	AGG	atg	Hypothetical novel protein		
gp76	58766	59218	-	tcaaccaagaaagat GGT aagtaaa	GGT	atg	Hypothetical novel protein		
gp77	59220	59972	-	acaaaatgccga AGGAG cgtgccaa	AGGAG	gtg	methyltransferase	IPR029063	
gp78	59969	60580	-	agttttttcaggc AGGA aacaaaaa	AGGA	atg	Hypothetical novel protein		
gp79	60717	60908	-	aatcaacgcatagaaagatttcacc	None	gtg	Hypothetical novel protein		
gp80	61285	61467	-	ctgagattgaaaa GAGG attttgtg	GAGG	atg	Hypothetical novel protein		
gp81	61467	64058	-	taatcaacactagac AGGA aatctc	AGGA	atg	von Willebrand factor (vWF)	IPR002035	
gp82	64180	66006	-	tcactgtaaagg GGA ttctgagaaa	GGA	atg	ATPase	IPR011704, IPR027417	
gp83	66915	67247	-	cagattgaccaggcac GGG ggatta	GGG	ttg	Hypothetical novel protein		
gp84	67394	68287	-	agaatccacagac AGG gcagagaaa	AGG	atg	Hypothetical novel protein		
gp85	68676	68852	-	gcataaaaccatgctca GAG attcc	GAG	atg	Hypothetical novel protein		1 tmd
gp86	68872	69378	-	aacactcacagac AGGA ctagaaaa	AGGA	atg	Hypothetical novel protein		
gp87	69495	69677	-	cagcacagacaggac AGGA ttaatc	AGGA	atg	Hypothetical novel protein		
gp88	71493	73511	-	actcacagaacggcag GGA taatcg	GGA	gtg	DNA primase	IPR002694	
gp89	73515	74006	-	aaactagcgagaa AGGA cataaaac	AGGA	atg	Hypothetical protein		
gp90	74035	74793	-	cccggcgcagac AGGAG atataggt	AGGAG	atg	Hypothetical protein		

Supplementary Table S3. Predicted proteins, gene starts and annotations of phage GordDuk1.

Protein name	Start	Stop	Strand	Upstream Sequence	S-D	Start Codon	Predicted product	Evidence	TMHMM
gp01	393	2012	+	ctggcactgtgctagcgt GGT ccac	GGT	atg	Hypothetical protein		
gp02	2013	5081	+	ttccgacgtt GAGG aagtgttctaa	GAGG	atg	TerL		
gp03	5216	7087	+	tgtcccgaacaca AGGAG aaatgta	AGGAG	atg	DUF935 like protein	IPR009279	
gp04	7143	7361	+	cctggggatatat GGA ctagaccat	GGA	atg	Hypothetical novel protein		
gp05	7392	8558	+	cagacaatcagta AGGT ggttaact	AGGT	atg	Hypothetical protein		
gp06	8573	9037	+	ttactctgtaag GGAG aaatagaaa	GGAG	atg	Hypothetical protein		
gp07	9050	10240	+	cacctttaagta AGGAG cctagaaa	AGGAG	atg	major capsid protein	IPR005564	
gp08	10273	10563	+	tcaaaccaga AGGA agtgaaatatc	AGGA	atg	Hypothetical novel protein		
gp09	10685	11218	+	aattcgagcggaaa GGGG cgggcga	GGGG	atg	Hypothetical protein		
gp10	11215	11703	+	cgcctgctccgcc GGAGGT tattcc	GGAGGT	gtg	Phage morphogensis protein	IPR006522	
gp11	11700	12203	+	aaacgtctattttt AGGA agctaaa	AGGA	gtg	Hypothetical protein		
gp12	12219	12524	+	gtttgagtaggaa AGGA acgagcaa	AGGA	atg	Hypothetical novel protein		
gp13	12536	13561	+	ctgacgagaacta AGGAG taataaa	AGGAG	atg	Hypothetical protein		
gp14	13708	14313	+	catcaagcctagaa AGG gcaataag	AGG	atg	pre-TMP frameshift protein		
gp15	13708	14573	+	catcaagcctagaa AGG gcaataag	AGG	atg	pre-TMP frameshift protein		
gp16	14680	24498	+	caaccttca AGGA tattgagtccat	AGGA	atg	Tape measure protein	IPR010090	7 tmd
gp17	24501	27080	+	ttcttggactggc AGGAG cttagaa	AGGAG	atg	Hypothetical protein		
gp18	27100	28833	+	atttaagtagagaaa GAG aagggca	GAG	atg	Hypothetical protein		
gp19	28830	30677	+	gaaacggaaagc GAG ttactaagct	GAG	atg	Hypothetical protein		
gp20	30681	31718	+	gtggtctagcgaaac GGA atgagtc	GGA	atg	Hypothetical novel protein		
gp21	31740	32123	+	ctagatttaatca AGGAG aaataaa	AGGAG	atg	Hypothetical protein		
gp22	32200	32901	+	catgaactaacgat AGGA tgagcgc	AGGA	atg	lysin	IPR013781	see text
gp23	32912	33277	+	ggaactcttgacta AGGAG aatgct	AGGAG	atg	Hypothetical novel protein		
gp24	33277	33933	+	atatgctaggaa AGGT tgtaggcta	AGGT	atg	LysA	IPR002502	see text
gp25	33935	34201	+	cagcttatgggaacgcg GGA ctgat	GGA	atg	Hypothetical novel protein		
gp26	34206	34574	+	gaaaacaccga AGGAG aataagatc	AGGAG	atg	LysA		
gp27	34584	35030	+	cagcaagattaagt GAGG aatgttt	GAGG	atg	holin		4 tmd
gp28	35030	35569	+	atcctgacaag GGA atcaccggata	GGA	atg	DNA methylase	IPR029063	
gp29	35694	35903	+	agaacgctgggaatc GGT aacgggc	GGT	atg	Hypothetical protein		
gp30	35900	36391	+	atgaccattgcctt GAGG attggat	GAGG	ttg	holin		4 tmd
gp31	36394	36792	+	ttaaacaggaaga AGG cgagtaaga	AGG	atg	holin		1 tmd
gp32	36838	37167	+	tcagctagcactc AGGAG agagcaa	AGGAG	atg	Hypothetical novel protein		
gp33	37164	37949	+	agcaagcagga AGGT tcattttcac	AGGT	atg	LysB	IPR029058, IPR013228, Hhpred: 1q66_A Acetyl xylan esterase I, 100.0; 4psc_A Carbohydrate esterase 100.0	
gp34	38052	38405	-	cgaatttacatgta AGGAG tgagaa	AGGAG	atg	Hypothetical novel protein		
gp35	38409	38564	-	tgatttcccggacta AGGA tcataa	AGGA	atg	Hypothetical novel protein		
gp36	38574	38858	-	tataagcac GGA cgaaagttctgaa	GGA	atg	Hypothetical novel protein		
gp37	38860	39399	-	cggaaagaatctttaatt ggg gcga	GGG	atg	Hypothetical protein		
gp38	39363	39689	-	ctcagactcagtatgatctagggat	None	atg	Hypothetical novel protein		
gp39	39736	40161	-	gcatttagga AGGA ttcaaaagtag	AGGA	atg	Hypothetical protein		
gp40	40154	40336	-	GAG taaacaagaaagctactaaact	GAG	gtg	Hypothetical novel protein		2 tmd
gp41	40333	40656	-	tctaatcctatgccaagtgattcag	None	atg	Hypothetical noval protein		
gp42	40643	41470	-	ttaagcaga AGG gccagtaataatt	AGG	atg	thymidylate synthase ThyX	IPR003669	
gp43	41476	42666	-	gattgatttgga GAGG ggaaatgaa	GAGG	gtg	Hypothetical protein		
gp44	42663	42833	-	accaaag AGGA ttggaattacgaac	AGGA	atg	Hypothetical novel protein		
gp45	42906	43070	-	caggcgtgaattaca GGGG gcagta	GGGG	atg	Hypothetical novel protein		
gp46	43070	43309	-	acaagaattagct AGGAG aaaagca	AGGAG	atg	Ferritin-related protein	IPR012347	
gp47	43320	43538	-	c GGT gattctcctaaattcttccaa	GGT	atg	Transcription factor WhiB	IPR003482	
gp48	43604	43852	-	taacgagtggta AGGA ttttagata	AGGA	atg	Hypothetical novel protein		
gp49	43865	44419	-	tcttgattacagtgatt GGT gcaat	GGT	atg	RusA-like ribonuclease	IPR008822	
gp50	44416	44667	-	gagatttgcggagaga AGGT gaatg	AGGT	atg	Hypothetical novel protein		1 tmd
gp51	44645	44863	-	tgtcaatcaacatg GGAG ttaagta	GGAG	atg	Hypothetical novel protein		
gp52	44863	45054	-	gggcaaa GAG aaagcgaactgggcg	GAG	atg	Hypothetical novel protein		2 tmd
gp53	45051	45203	-	atgcaccaaatgcc GAG aaggaata	GAG	atg	Hypothetical novel protein		
gp54	45203	45511	-	caggtagaaatcga AGGAG cataaa	AGGAG	atg	Hypothetical novel protein		
gp55	45513	46649	-	gaaattggaatt GGAG taattggat	GGAG	atg	DNA polymerase		
gp56	46656	46778	-	cctgtttattgagt GAGG aatcgaa	GAGG	atg	Hypothetical novel protein		
gp57	46788	47378	-	aagaaaccggagaa GAGG attaatc	GAGG	atg	ribonuclease	IPR012337, IPR013520, IPR006055	
gp58	47381	47638	-	aggaaaaagaaaa GAGG aataagaa	GAGG	atg	DNA binding domain protein	IPR011991, IPR009061, IPR010093	
gp59	47635	49176	-	tgaataagaa AGGAG cacggttaat	AGGAG	atg	Helicase	IPR014001, IPR001650, IPR027417	
gp60	49195	49572	-	aagatttatcgagtagt GGA ggttc	GGA	atg	Hypothetical novel protein		
gp61	49553	49780	-	cccaagcatg AGGA cctgatctaaa	AGGA	atg	Hypothetical novel protein		
gp62	49782	50228	-	aattcttcaagac AGGA tcaagaaa	AGGA	atg	DUF732 like protein	IPR007969	
gp63	50549	51214	-	aaaatcgaaaca AGGAG aaaacaaa	AGGAG	atg	Hypothetical protein		
gp64	51370	51660	-	cttaaacccagtagcg GGGT ctaga	GGGT	gtg	Hypothetical novel protein		
gp65	51662	52528	-	aagttcggaagattt GGAGG aactg	GGAGG	atg	Hypothetical novel protein		
gp66	52458	52970	-	ctagtattttggaat GGT gagaaaa	GGT	atg	Hypothetical novel protein		
gp67	52963	53289	-	atcaactgtttggca AGGAG ctaaa	AGGAG	gtg	Hypothetical novel protein		
gp68	53286	53570	-	tcgagcaattccggt GGT gaattaa	GGT	atg	Hypothetical novel protein		
gp69	53571	53867	-	gaagatcaggaagagc AGGA aatca	AGGA	atg	Hypothetical novel protein		
gp70	53860	54210	-	ggcgcac GAGG cggtgaatttctaa	GAGG	atg	Hypothetical novel protein		
gp71	54211	55008	-	cactaactcacagac AGGA aaagaa	AGGA	atg	integrase	IPR011010, IPR002104, IPR013762	
gp72	55172	55552	-	caaaataccgac AGGAG aacgaaaa	AGGAG	gtg	Hypothetical protein		
gp73	55549	55878	-	aaaatcaagagacag GGA tttatca	GGA	atg	Hypothetical novel protein		
gp74	56008	56874	-	tagatatgatagaaac GGAG taaaa	GGAG	atg	Hypothetical novel protein		
gp75	56877	57257	-	tttattatgattaagt GGT cgggat	GGT	atg	Hypothetical novel protein		
gp76	57407	57598	-	gaagaggctcg GGAGGT tcttgcaa	GGAGGT	atg	Hypothetical novel protein		
gp77	57591	58301	-	atcagagaaatgttct GGA acgata	GGA	atg	Hypothetical novel protein		
gp78	58301	58543	-	gaccgaggaaagtag GGAG aaaaca	GGAG	gtg	Hypothetical novel protein		
gp79	58540	58707	-	aaaagcacaggaac AGGT acaccct	AGGT	atg	Hypothetical novel protein		
gp80	58704	58922	-	gaccatgcggaatt GGAG attgcaa	GGAG	atg	Hypothetical novel protein		
gp81	58886	59275	-	atcgcgttattg GGA tgactaccaa	GGA	atg	Mycobacteriophage protein	IPR019627	
gp82	59335	59550	-	tcgagcgatacgtcaagt AGG ggaa	AGG	atg	Hypothetical novel protein		
gp83	59556	60008	-	tcaaccaaga AGG gcggtaagtaaa	AGG	atg	Hypothetical novel protein		
gp84	60010	60753	-	acaaaatgccga AGGAG cgtgtcaa	AGGAG	gtg	methyltransferase	IPR029063	
gp85	60750	61370	-	acacagcacagac AGGA aataaaaa	AGGA	atg	Hypothetical novel protein		
gp86	61497	61688	-	aatcaacgcatagaaagatttcacc	None	gtg	Hypothetical novel protein		
gp87	62246	64879	-	taatcaacacgagac AGGA aatctc	AGGA	atg	von Willebrand factor (vWF)	IPR002035	
gp88	65001	66848	-	tcactgtaaa GGGG attctgagaaa	GGGG	atg	ATPase	IPR011704, IPR027417	
gp89	67760	68083	-	caggcacggg GGA ttattgcgctct	GGA	atg	Hypothetical novel protein		
gp90	68227	69111	-	cggaatcacagac AGGA cagagaaa	AGGA	atg	Hypothetical novel protein		
gp91	69499	69690	-	aactagcacagac AGGA ttaaagcc	AGGA	atg	Hypothetical novel protein		1 tmd
gp92	69710	69964	-	aacactcacagac AGGA ctagaaaa	AGGA	atg	Hypothetical novel protein		
gp93	70088	70270	-	accagcacagac AGGA ttagaaaac	AGGA	atg	Hypothetical novel protein		
gp94	70375	70752	-	atcaacagagac AGGA caggacatt	AGGA	atg	Hypothetical novel protein		
gp95	72598	74616	-	actcacagaacggcag GGA taatcg	GGA	gtg	DNA primase	IPR002694	
gp96	74620	75111	-	aaactagcgagaa AGGA cataaaac	AGGA	atg	Hypothetical protein		
gp97	75140	75898	-	cccggcgcggac AGGAG atataggt	AGGAG	atg	Hypothetical protein		

Supplementary Table S4. Predicted proteins, gene starts and annotations of phage Gsput1.

Protein name	Start	Stop	Strand	Upstream Sequence	S-D	Start Codon	Predicted product	Evidence	TMHMM
gp01	149	307	+	cctgtgttgatctca GGGG cgcgtt	GGGG	gtg	Hypothetical novel protein		
gp02	471	719	-	acgctaaaggcgta GAG tcatacac	GAG	atg	Hypothetical novel protein		
gp03	1005	1634	+	atttcgtatcaacac GAGG acatg	GAGG	atg	LysB	IPR029058, IPR000675	1 tmd
gp04	1649	1978	+	acacctactagacgaa GGGG gcacc	GGGG	gtg	Hypothetical conserved protein		
gp05	1941	3626	+	acagaacaggtgaa GGGG gcgctga	GGGG	gtg	TerL	IPR005021	
gp06	3629	4933	+	acgactatctgatatt GGAG tgaca	GGAG	gtg	Portal protein	IPR006427, IPR006944	
gp07	4923	5735	+	gagccggatgac GAG agcgggaacg	GAG	atg	Prohead protease	IPR006433, IPR001847	
gp08	5827	7071	+	atacgggcgaacgaa GGGG caacaa	GGGG	atg	Phage capsid protein	IPR006444, IPR024455	
gp09	7154	7360	+	ttcgtgcgtta GGGG acgaatcacg	GGGG	gtg	Hypothetical novel protein		
gp10	7323	7862	+	gctcgcccgaatccgacagtgacac	None	atg	Hypothetical conserved protein		
gp11	7862	8218	+	ttgacccgttccgggc GGT ggtctg	GGT	atg	Hypothetical conserved protein		
gp12	8215	8484	+	aggccgagctga AGGT gattacagg	AGGT	atg	Hypothetical conserved protein		
gp13	8489	8701	+	cgggccgcaagg GGAGGT tgacgac	GGAGGT	atg	Hypothetical novel protein		
gp14	8688	9059	+	gccgcaacca AGG gcgacgcgggcg	AGG	atg	Hypothetical novel protein		
gp15	9141	9716	+	ttcgtgtgccaatga AGGAG agagc	AGGAG	atg	Hypothetical conserved protein		
gp16	9894	10244	+	caacccacggaatc AGGA aacacac	AGGA	atg	pre-TMP frameshift protein		
gp17	9894	10591	+	gcggggcga GGT ctcactacgcaag	GGT	ttg	pre-TMP frameshift protein		
gp18	10609	14904	+	gattataggtaaagc GGGT gatcgc	GGGT	atg	tape measure protein	IPR010090, IPR008258, IPR023346	
gp19	14904	15749	+	tcgcacatacgaaggc AGGT gtgtg	AGGT	atg	phage tail protein	IPR008841	
gp20	15750	17078	+	gccgcgttaccga AGG gcttggtaa	AGG	atg	Hypothetical conserved protein		
gp21	17048	18142	+	ttcggcgcgcaa AGGA tctcgaaaa	AGGA	atg	Endolysin	IPR002502	
gp22	18136	18396	+	tagcgcacggca AGGT gtcgggcga	AGGT	atg	Holin		2 tmd
gp23	18403	18867	+	tgccgagccttcc GAG tagtcgggc	GAG	gtg	Hypothetical novel protein		1 tmd
gp24	18864	19922	+	agcacgagggacttca GGGG atcgg	GGGG	atg	Hypothetical conserved protein		
gp25	19933	21690	+	ccggttgtgaact AGGAG agatcgc	AGGAG	atg	galactose binding protein	IPR008979	
gp26	21693	22775	+	taacatttctc GGGG cggcgtaagt	GGGG	atg	Hypothetical conserved protein		
gp27	22779	23123	+	atggatcaaggc GAGG cagtgactt	GAGG	atg	Hypothetical conserved protein		
gp28	23361	24542	-	ggctacgaact GGA atggtgagact	GGA	gtg	Integrase	IPR011010, IPR023109, IPR013762, IPR002104	
gp29	24547	24849	-	cccgtaagcgcgtaacgtgcctagt	None	gtg	DNA binding protein	IPR009061, IPR011991	
gp30	24935	25768	+	ccgctaacggtgta GGGT ctcattc	GGGT	atg	heat shock protein	IPR001305	
gp31	25835	26551	-	cgcaagttcgaa AGGA tctccttag	AGGA	ttg	Hypothetical novel protein		7 tmd
gp32	26815	27093	+	cgtgtc GGGT tgacttgccgcacat	GGGT	gtg	DNA binding protein	IPR010982	
gp33	27090	27284	+	acgccccgacacttcggc AGG cgtc	AGG	gtg	DNA binding protein	IPR009061	
gp34	27343	27729	+	caggattcatga GAGG aaaaacgaa	GAGG	atg	Hypothetical novel protein		
gp35	28171	28320	+	cacacgaaagagata GGGG cacagc	GGGG	atg	Hypothetical novel protein		
gp36	28403	28834	+	ccaaccaattga GAGG gactgttca	GAGG	gtg	Hypothetical novel protein		
gp37	28831	29070	+	ccttcgagctgatc GAGG cggcgct	GAGG	gtg	Hypothetical novel protein		
gp38	29067	29330	+	ccccgcaactcgcggc GGT ggccga	GGT	gtg	Hypothetical novel protein		
gp39	29451	30368	+	tcgtaactagagctat GGGG tactg	GGGG	gtg	Hypothetical conserved protein		
gp40	30337	30849	+	ggcgacgagtg GGA ccactacgagc	GGA	atg	Hypothetical conserved protein		
gp41	30846	31469	+	atcaatcagcaa AGGAG cgaaggcg	AGGAG	ttg	DNA recombination protein	IPR007499	
gp42	31462	31854	+	gaagcattggaa GGGG cacaaccga	GGGG	atg	Hypothetical novel protein		
gp43	31851	32147	+	tgacgcttgcgggac GGGG gcaggc	GGGG	gtg	Hypothetical novel protein		
gp44	32260	32490	+	GGT gcatcaccgacgcccgcgaggc	GGT	atg	Hypothetical conserved protein		
gp45	32516	33187	+	gccgaatttttggc GGT cgctcgat	GGT	atg	Hypothetical conserved protein		
gp46	33194	34504	+	gcgtattcagttcgatt GAG ggtga	GAG	atg	DNA helicase		
gp47	34521	34976	+	caactctaaca AGGAG attcaacgc	AGGAG	atg	DNA binding protein	IPR012340, IPR000424, IPR011344	
gp48	35124	35474	+	ccgaaggc GGA cattgcctaatcgc	GGA	atg	endonuclease	IPR007560	
gp49	35471	35674	+	tggcgcgagcc GGA tacggggaagc	GGA	atg	Hypothetical novel protein		
gp50	35671	35868	+	tcacagacgaa GGGG cgtgggcgga	GGGG	atg	Hypothetical novel protein		
gp51	35855	36355	+	gagtcgggttggc AGGT ggtcacgc	AGGT	ttg	Hypothetical conserved protein		
gp52	36371	36856	+	catcatct AGGAGG cgcgcgtgaaa	AGGAGG	atg	HNH endonuclease	IPR002711, IPR003615	
gp53	36846	37109	+	gacctactgatttcggc GGT gcgga	GGT	atg	Hypothetical conserved protein		
gp54	37265	37585	+	caactacccga GAGG gattcaaaca	GAGG	gtg	Hypothetical novel protein		
gp55	37586	37813	+	gaggccagcgggcaa GGGG agttaa	GGGG	ttg	glutaredoxin	IPR002109, IPR012336, IPR011909	
gp56	38093	38275	+	caagcatcgga AGG caatcgcggca	AGG	atg	Hypothetical novel protein		
gp57	38283	39401	+	gtctgtaatcgaaagt AGGT gcgct	AGGT	atg	phosphatase	IPR029052	
gp58	39429	39635	+	ctgcgccgaatcactgaa AGG ttga	AGG	atg	Hypothetical novel protein		
gp59	39616	40152	+	gactacgtgacg GGAG aaattgcgc	GGAG	gtg	Hypothetical novel protein		
gp60	40139	40258	+	ctggacaacctgaa GGGG atcactc	GGGG	gtg	Hypothetical novel protein		
gp61	40315	40578	+	tcaacacttgatgga AGGT cattgt	AGGT	gtg	Hypothetical novel protein		
gp62	40603	40947	+	aa GGGG cgcaagcatttacaaactc	GGGG	gtg	Hypothetical conserved protein		
gp63	40934	41092	+	ggcgcatggctcgat AGGT ggcagc	AGGT	gtg	Hypothetical novel protein		
gp64	41089	41247	+	tcaccgacctactac GGGG gatcaa	GGGG	atg	Hypothetical novel protein		
gp65	41393	42007	+	atcaacacattcgga AGGT gtgcaa	AGGT	gtg	Hypothetical conserved protein		
gp66	42004	42306	+	cggcggtagcgcaga AGGT gctcgc	AGGT	gtg	Hypothetical novel protein		
gp67	42303	42482	+	aagtcaacgcaggga AGGT gctcgc	AGGT	atg	Hypothetical novel protein		
gp68	42469	42624	+	ctgattgtggaa AGGA aacccacgc	AGGA	atg	Hypothetical novel protein		
gp69	42621	42830	+	attacgggcctccggt GGT gtggct	GGT	gtg	Hypothetical novel protein		
gp70	42827	43000	+	aggaactactcagga AGGT atgccc	AGGT	atg	Hypothetical novel protein		
gp71	43223	43483	+	gatcaaagac GGAGG gaacgaccag	GGAGG	gtg	Hypothetical novel protein		
